# Intention tremor and deficits of sensory feedback control in multiple sclerosis: a pilot study

**DOI:** 10.1186/1743-0003-11-170

**Published:** 2014-12-19

**Authors:** Megan Heenan, Robert A Scheidt, Douglas Woo, Scott A Beardsley

**Affiliations:** Department of Biomedical Engineering, Marquette University, Milwaukee, WI USA; Physical Medicine and Rehabilitation, Northwestern University Feinberg School of Medicine, Chicago, IL USA; Department of Neurology, Medical College of Wisconsin, Milwaukee, WI USA; Clinical and Translational Science Institute, Medical College of Wisconsin, Milwaukee, WI USA; Department of Neurology, Fairfield Healthcare Professionals Neurology, Lancaster, OH USA; Department of Biomedical Engineering, Boston University, Boston, MA USA

**Keywords:** Multiple sclerosis, Intention tremor, Dysmetria, Sensorimotor, Motor control, Neuromotor control

## Abstract

**Background:**

Intention tremor and dysmetria are leading causes of upper extremity disability in Multiple Sclerosis (MS). The development of effective therapies to reduce tremor and dysmetria is hampered by insufficient understanding of how the distributed, multi-focal lesions associated with MS impact sensorimotor control in the brain. Here we describe a systems-level approach to characterizing sensorimotor control and use this approach to examine how sensory and motor processes are differentially impacted by MS.

**Methods:**

Eight subjects with MS and eight age- and gender-matched healthy control subjects performed visually-guided flexion/extension tasks about the elbow to characterize a sensory feedback control model that includes three sensory feedback pathways (one for vision, another for proprioception and a third providing an internal prediction of the sensory consequences of action). The model allows us to characterize impairments in sensory feedback control that contributed to each MS subject’s tremor.

**Results:**

Models derived from MS subject performance differed from those obtained for control subjects in two ways. First, subjects with MS exhibited markedly increased visual feedback delays, which were uncompensated by internal adaptive mechanisms; stabilization performance in individuals with the longest delays differed most from control subject performance. Second, subjects with MS exhibited misestimates of arm dynamics in a way that was correlated with tremor power. Subject-specific models accurately predicted kinematic performance in a reach and hold task for neurologically-intact control subjects while simulated performance of MS patients had shorter movement intervals and larger endpoint errors than actual subject responses. This difference between simulated and actual performance is consistent with a strategic compensatory trade-off of movement speed for endpoint accuracy.

**Conclusions:**

Our results suggest that tremor and dysmetria may be caused by limitations in the brain’s ability to adapt sensory feedback mechanisms to compensate for increases in visual information processing time, as well as by errors in compensatory adaptations of internal estimates of arm dynamics.

**Electronic supplementary material:**

The online version of this article (doi:10.1186/1743-0003-11-170) contains supplementary material, which is available to authorized users.

## Introduction

Accurate arm and hand movements are the key to performing many daily tasks, but in individuals with Multiple Sclerosis (MS), the processes that control these movements are disrupted due to demyelination of the axonal projections that transmit information within and between brain areas. Upper extremity motor dysfunction in MS most often manifests as kinetic tremor (uncontrolled rhythmic motion of the joints during goal-directed movements) or dysmetria (a lack of coordination of movements typified by the under- or overshoot of the intended position of the hand or arm). Up to seventy-five percent of individuals with MS experience tremor in the arms and hands, with as many as 27% of those reporting tremor-related disability [1–4]. Drug therapies [5–9] and surgical treatments [2, 10–12] can mitigate some effects of tremor, although their effectiveness decreases over time [13] (for review see [14]). Recently, rTMS has been used to reduce tremor [15], however, the effects are short-lived.

Because neural lesions that develop in MS are distributed throughout the central nervous system, similar movement deficits (i.e. tremor) may result from differing impairments in the sensory feedback control pathways. Consequently, the specific neuroanatomical etiology of tremor and dysmetria remain unclear. Tremor and dysmetria are most often associated with lesions in the cerebellum and/or the thalamic nuclei, suggesting impairment of the cortico-cerebellar sensorimotor control loops used for the planning and adaptive control of movement [16, 17]; for review see Koch, et al. [4]. Recent studies also implicate impairments of the predictive mechanisms used to guide movement and/or degradation of the sensory information upon which such predictions are based, including impairment in sensory transmission of information, which is lengthened in those with MS [1, 2, 18–21]. The many-to-one mapping of the source of impairment onto clinical symptoms poses significant challenges for developing effective therapies. For example, a therapy designed to compensate for one patient’s dysmetria caused by increased sensory processing delays may not be effective for another patient whose dysmetria is due to impaired prediction of limb dynamics.

Exercise-based rehabilitation strategies can improve posture and movement control over the short-term, but they have had less success in achieving significant long-term reductions in motor incoordination generally, and tremor or ataxia specifically (for review see Brown and Kraft 2005) [22–28]. Recent studies by Feys and colleagues have demonstrated that motor performance in MS can be enhanced by exploiting the inherent adaptability of sensorimotor control mechanisms. For example, Feys, et al. (2001, 2006) have found that altering visual feedback information can reduce intention tremor and improve performance on functional tasks [22, 23]. Other studies that use robotic devices to introduce mechanical perturbations and practice correcting erroneous movements, can improve movement control, reduce tremor, and improve coordination [27–29]. However, the mechanisms by which these approaches are able to improve functional performance remains unclear.

In this study, we describe a systems-level computational model and an experimental technique that parameterizes subject-specific deficits in sensory feedback control of the elbow joint [30–33] in individuals diagnosed with MS. We used this approach to test the hypothesis that tremor in MS results from subject-specific impairments in the adaptive feedback processes that guide movement. Specifically, we fit the parameters of a dual-feedback, sensorimotor control model to the kinematic data obtained from each subject’s responses to perturbations during a series of continuous elbow flexion/extension tasks [31, 33]. We compared the parameters obtained from subjects with MS to those of age- and gender-matched, healthy control subjects to identify the sensory and/or motor processes affected by MS, and the extent to which they correlate with intention tremor. Future studies could use this approach to characterize changes in sensory feedback control induced by therapeutic intervention to advance understanding of how to best mitigate individuals’ deficits of motor function as they evolve with progression of the disease.

## Methods

### Subjects

Sixteen subjects participated in the study. Eight subjects had clinical diagnoses of MS and exhibited mild to severe tremor (ages 25–68 years old, 6 female, 7 right-handed). Eight healthy participants served as age- (±7 yrs) and gender-matched control subjects (ages 26–61 years old, 6 female, 8 right-handed). All participants provided written, informed consent in accordance with the Declaration of Helsinki and as approved by institutional review boards at Marquette University and the Medical College of Wisconsin.

Subjects with MS were assessed clinically in a session conducted at the Medical College of Wisconsin prior to participating in the primary study (Table 1). Disease duration ranged from 6 to 30 years. Six subjects with MS had received disease-modifying therapy with either an immune-modulator or immunosuppressant, with four subjects continuing therapy at the time of the study. Severity of disability on the Expanded Disability Status Scale (EDDS) ranged from 1 to 7 (out of 10), with three subjects confined to a wheelchair. All of the subjects exhibited motor strength in the upper extremities of 4 or greater on the Medical Research Council system of grading, and all demonstrated normal tone and normal proprioceptive sensation on exam. Visual acuities were 20/40 or better in all subjects. Scores on the Ataxia Scale for Dysmetria and the Tremor Assessment Scale ranged from 1 to 3 (out of 4).

**Table 1:** **Clinical characteristics of MS subjects including disease type (RR: relapsing remitting, PP: primary progressive, SP: secondary progressive, PR: progressive relapsing), expanded disability status scale (EDSS), tremor and ataxia scores obtained during a separate clinical evaluation**

Subject #	Age	Gender	Dominant hand	MS Type	EDSS	Tremor score*	Ataxia score*	NHPT* (sec)
**1**	45	F	R	RR	2	1	1	27.9
**2**	57	F	R	PP	7	1	1	18.9
**3**	31	F	R	RR	2	2	1	27.0
**4**	29	F	R	SP	7	2	2	81.6
**5**	55	F	R	-	6	2	2	DNC
**6**	25	M	R	PR	7	3	2	75.1
**7**	41	M	R	RR	6	3	2	77.2
**8**	68	F	L	RR	1	3	3	141.0

Nine Hole Peg Test (NHPT) times were obtained the day of testing (* indicates right hand only; DNC: did not complete in time allotted).

### Sensory feedback control model

**Figure 1:**
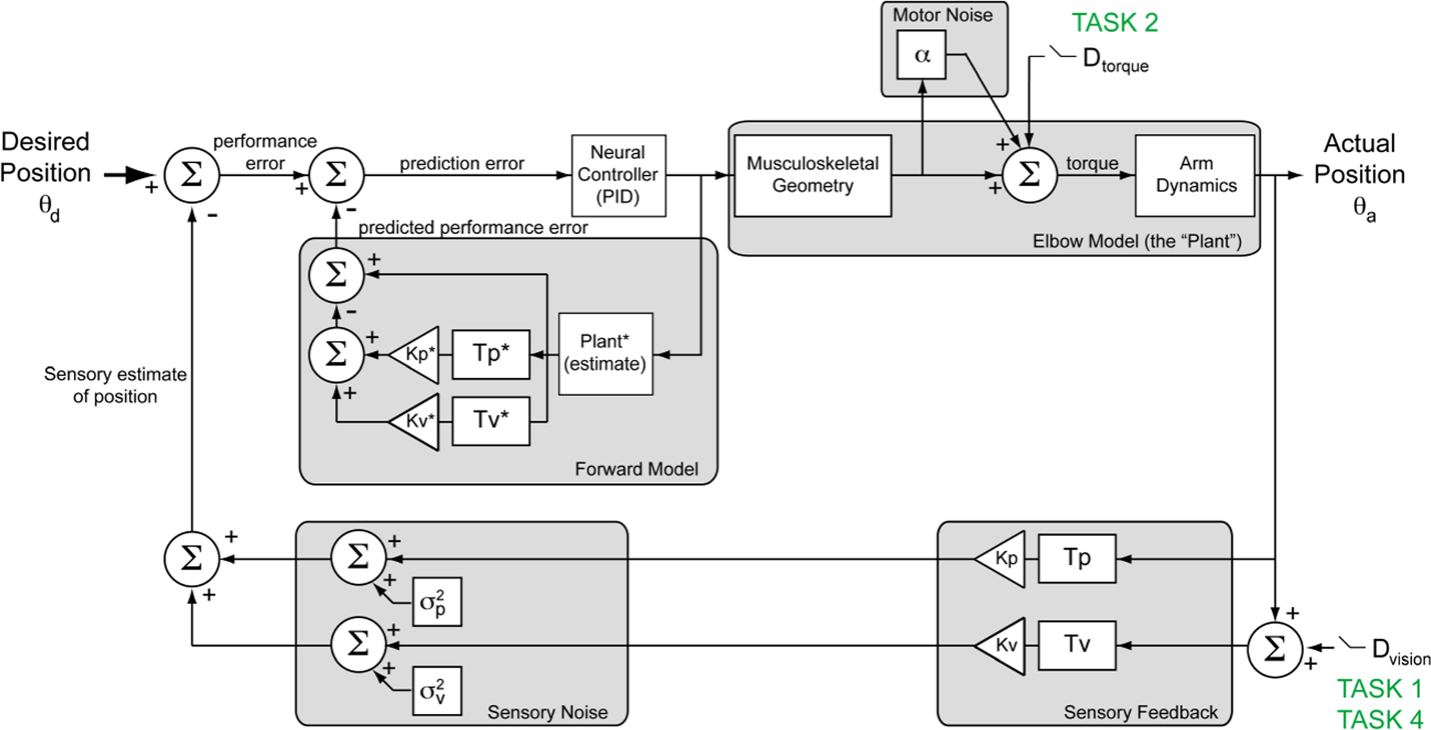
**Multisensory feedback model of sensorimotor control.** The model consists of a feed-forward motor control path and three nested feedback paths. The outermost feedback path accounts for sensory (visual and proprioceptive) feedback. In the forward path, neural processing associated with the correction of state errors (i.e. the difference between desired, θ_d_, and observed, θ_a_, elbow angles) is modeled generically by a PID controller (inverse model) containing separate proportional, integral, and derivative gains. Motor noise in the generation of torques is modeled by a multiplicative noise (α). Corrective torque is converted to angular position of the arm using a 2nd order model characterizing the inertia, viscosity, and stiffness about the elbow. In each branch of the outer-most feedback path, arm position is delayed (*T*) and weighted (*K*) to provide a combined sensory estimate of arm position. The forward model provides predictive compensation of the arm dynamics and delays via the inner feedback loops. D_ext_ denotes external perturbations applied to the perceived visual and/or proprioceptive (i.e. physical) feedback of arm position.

Sensory feedback control includes adaptive feedforward and feedback mechanisms. Based on the work of Peterka [30], and McRuer [34, 35], we have developed a closed-loop model of sensory feedback control during goal-directed movement and have used it previously to describe sensorimotor responses to environmental perturbations and distortions of visual feedback (Figure 1) [31, 33]. In the current study, we use the sensory feedback control model to examine how MS impacts feedback control. In the model, angular position error of the elbow joint (i.e. *performance error*) is calculated as the difference between desired position (θ_d_) and the weighted sum of visual and proprioceptive estimates of the actual arm position (θ_a_) [30–33, 36]. The weights of the visual and proprioceptive paths are represented by K_v_ and K_p_, respectively. Delays in visual and proprioceptive processing are modeled separately (T_v_ and T_p_ , respectively) to account for the overall response delay in each sensory path; these lumped-parameter terms combine feedback delays due to signal conduction and sensory information processing. In the forward path, actual performance error is compared to the predicted consequences of the intended action (i.e. the output of a forward model) to yield an instantaneous prediction error, which gives rise to a set of muscle activations through the action of a neural feedback controller, which for simplicity we model using a Proportional-plus-Integral-plus-Derivative (PID) controller, C(s):

1 $$C\left(s\right)=K_{d}s+K_{pr}+\frac{K_{i}}{s}.$$

This generic controller, which has previously been used to model movement control [30], contains separate derivative (K_d_) proportional (K_pr_) and integral (K_i_) gains to allow the controller to minimize transient response errors as well as steady state errors. The output of the controller is a scalar quantity representing the intended net muscle activations, which in turn act through the musculoskeletal geometry (muscle attachment points and moment arms) to give rise to a net torque applied to the physical plant (the forearm/hand pivoting about the elbow). Note that we have simplified our model of the physical plant by discounting muscle activation/contraction dynamics, which are assumed to be dominated by the second-order passive dynamics of the arm. We do, however, account for variations in muscle fiber recruitment [37] by reducing the precision of the intended torque by a multiplicative motor noise (α).

The arm’s dynamical response to the applied torque is estimated using a second-order model, P(s),

2 $$P\left(s\right)=\frac{1}{Js^{2}+Bs+K}$$

This model simulates the passive mechanical properties of the forearm and hand about the elbow via separate inertia (J), viscosity (B), and stiffness (K) terms.

The sensory feedback control model also includes an internal feedback path (referred to here as a forward model), which provides predictions of movement kinematics and the sensory consequences of those actions based on efference copy of the intended motor actions and internal estimates of the sensory gains (K_v_*, K_p_*), system delays (T_v_*, T_p_*), and limb dynamics (Plant* - Eq. 4). One important effect of the forward model is to compensate for the long-latency feedback loops (>100 ms) associated with sensory processing.

### Experiment setup

All subjects participated in a single, two-hour experimental session wherein they performed a series of five compensatory tracking tasks to characterize sensory feedback control about the elbow. Tasks and analysis are summarized in Table 2. All subjects also performed a spiral tracing task to quantify tremor frequency and amplitude [38]. Subjects with MS additionally performed the 9-hole peg test (9HPT) at the beginning of the experimental session for comparison with clinical assessments (9HPT, EDSS [39], ataxia and tremor scores [40]). Elbow angle and joint torque data collected during performance of four single-joint tracking tasks were used to obtain an individualized (best-fit) estimate of the sensory feedback control model depicted in Figure 1. Data collected from the fifth single-joint tracking task was used for model validation. The order of task presentation was counterbalanced across subjects. In order to account for potential task-related variations in subjects’ responses, all model parameters (aside from the physiological parameters: sensory delays and muscle noise) were measured simultaneously during a single compensatory tracking task performed in the presence of high-frequency position perturbations (“task 4”, described below).

**Table 2:** **Experimental tasks and analysis**

Task	Input	Parameter measured		Analysis method
Spiral tracing	Line tracing	*f*_*t*_ *, M*_*t*_	Tremor frequency and magnitude	Power spectrum analysis
Task 1	Low frequency visual perturbation	T_v_	Visual response delay	Cross correlation of subject response with input
		T_v_^*^	Predictive response delay	
Task 2	Low-frequency torque perturbation	T_p_	Proprioceptive response delay	Cross correlation of subject response with input
		T_p_^*^	Predictive proprioceptive response delay	
Task 3	Fixed levels of isometric torque	α	Multiplicative motor noise	Linear fit of variance vs. average torque
Task 4	High-frequency visual perturbation	J, B, K	Inertia, viscosity, stiffness of arm	Bootstrapped model fit to the subject’s measured frequency response function (FRF)
		J^*^, B^*^, K^*^	Predictive inertia, viscosity, stiffness of arm	
		K_v_, K_p_, K_v_^*^, K_p_^*^, K_d_, K_pr_, K_i_	Sensory and Controller gains	
Task 5	Visual offset	RMSE	Movement error	Kinematic analysis

* indicates predictive values.

**Figure 2:**
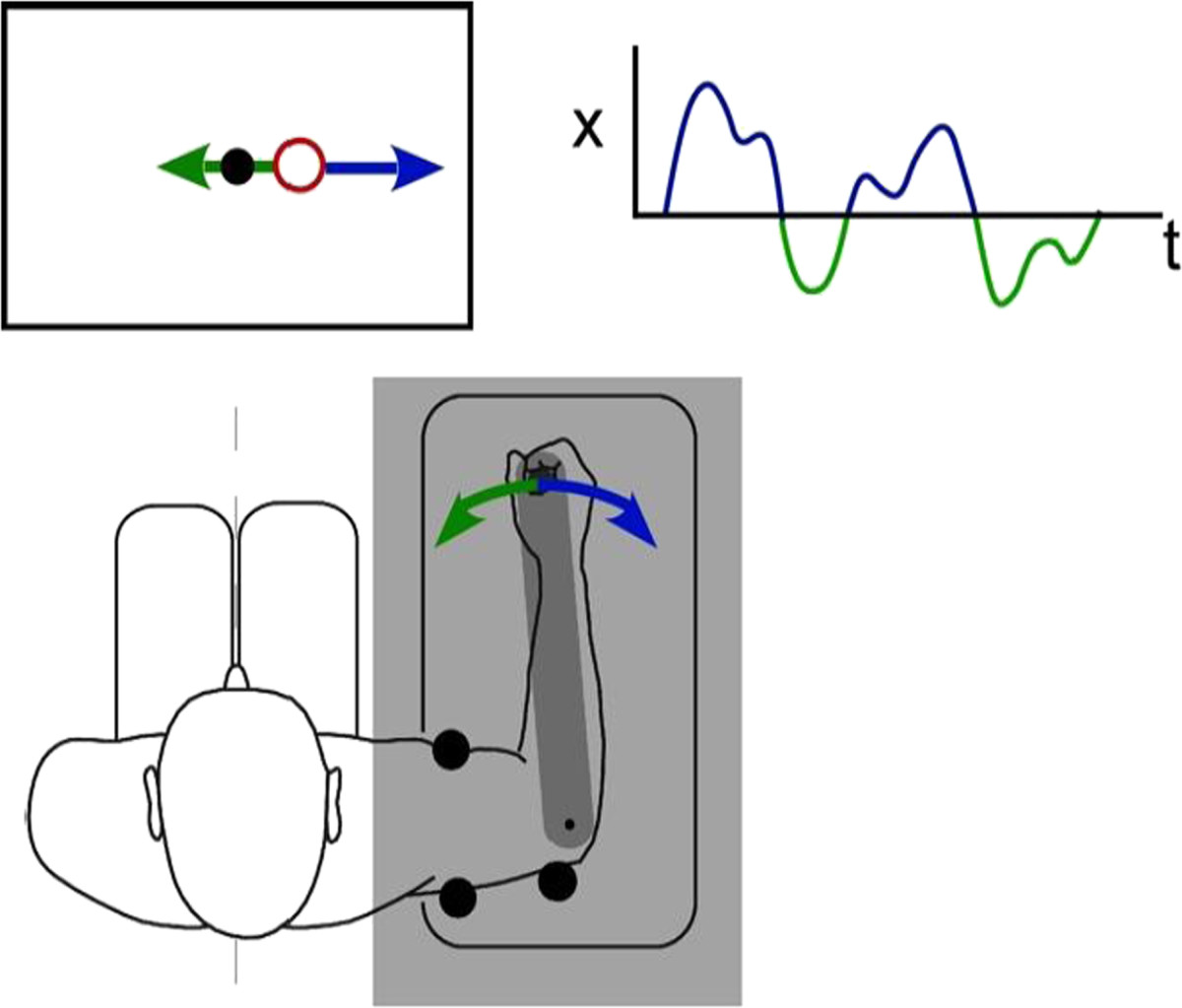
**Experimental setup.** Subjects held the handle of a 1-D manipulandum while seated in front of a computer display. The position of a cursor (red ring) was manipulated by rotating the manipulandum handle about the elbow joint. The cursor (or arm) was continuously perturbed (upper right inset) with a zero-mean, band-limited disturbance, and the subject was asked to compensate by bringing the cursor to a target (black circle) presented in the center of the display. The arm was occluded by an opaque screen (shaded region) so that the cursor provided the only visual cue of arm movement.

During single-joint tracking tasks, subjects held the handle of a 1-D robotic manipulandum with their right hand (Figure 2); the robot’s axis of rotation was aligned with that of the elbow joint such that the subjects’ arms were supported at an angle of approximately 90° of adduction. Details of the robot implementation and control can be found in [41]. Rotation of the manipulandum about the elbow (limited to ±40° relative to the sagittal plane) was yoked to the horizontal position of a cursor (a red ring) displayed on a 19-inch computer monitor. The monitor was placed perpendicular to the line of sight at a distance of 60 cm, which resulted in a cursor diameter of 0.67°. During the tasks, a stationary target (a black circle 0.33° in diameter) was also displayed on the screen. Direct view of the arm was blocked using an opaque barrier such that the cursor provided the sole visual cue of arm position. Rigid supports were placed on either side of the subject’s upper arm to minimize shoulder and/or upper arm movements.

Continuous, visual or torque perturbations were applied to the cursor or arm, respectively, during the 1-D target tracking tasks described below. Subjects were asked to compensate for low- or high-frequency perturbations (low-frequency: 0-1Hz, band-limited white noise; high-frequency: 0-10Hz, band-limited white noise, low-pass filtered at 1 Hz) by returning the cursor or arm back to the desired target location as quickly and accurately as possible. Subjects performed between 10 and 25 trials per task. Trial lengths varied by task and ranged from 8–32 seconds, with 15–30 seconds of rest between trials. During the rest period, the screen displayed the instruction “relax”. Two seconds before the start of the next trial, subjects were cued to “get ready”. The trial then began when the cursor and target appeared on the screen.

### Target tracking tasks

#### 2D spiral tracing task

A digitized spiral tracing task (adapted from Feys and colleagues) [38], was used to characterize the temporal frequency and signal power of each subject’s tremor. During the task, subjects used a digital pen to trace the line of an Archimedes spiral (center-out) overlaid on a Wacom digital tablet (12×18.2 inch drawing surface; Wacom Technology Corporation, Vancouver, WA). Throughout the task, subjects self-supported their arm against gravity and were instructed not to rest their arm or hand on the table while they traced the spiral in the transverse plane. Pen location data was collected at 200 samples/second in Matlab ver. 8.2 using the Cogent 2000 toolbox (Laboratory of Neurobiology, University College London, London, UK). The spiral was labeled with tick marks every 3 cm. To prevent the adoption of strategies that would compensate for tremor during the task, subjects were instructed to adjust their tracing speed according to a metronome such that they crossed one tick mark per beat, while keeping the movement as smooth as possible. Metronome speeds were adjusted based on the subject’s ability to maintain the target speed, and ranged from 60 bpm (3 cm/sec) to 240 bpm (12 cm/sec). Prior to the task, subjects performed five practice trials to familiarize themselves with the task and timing requirements. The first two practice trials were completed at the subjects’ self-selected speeds without the metronome. In the remaining practice trials, subjects performed the task with the metronome, starting at 60 bpm and increasing the metronome speed by an additional 40 bpm in each subsequent trial to identify a comfortable base speed. Following the practice trials, subjects completed ten “test” trials limited to 20 seconds each. During the first eight test trials, task difficulty was increased from the base speed on every second trial by incrementing the metronome speed an additional 40 bpm (2 cm/sec) until the subjects’ tracing speed fell below 90% of the metronome speed. During the last two trials, subjects were told to move as quickly as possible while accurately tracing the entire spiral.

#### 1-D target tracking

##### Tasks 1 and 2

**compensatory tracking with low-frequency perturbations** Visual *(T*_*v*_* , T*_v_*) and proprioceptive *(T*_*p*_* , T*_p_*) delays were characterized in separate compensatory tracking tasks. Subjects performed 10 trials per task and trial duration was 20 seconds in each case. In the first task (Task 1), visual response delays (*T*_*v*_* , T*_v_*) were characterized by applying continuous pseudorandom visual displacements (0.05 – 1 Hz; RMS = 10° visual angle) to the cursor position while subjects applied counter movements to the manipulandum so as to maintain the cursor on a stationary target presented at the center of the display.

In the second task (Task 2), proprioceptive response delays (*T*_*p*_* , T*_p_*) were characterized by applying continuous pseudorandom torque perturbations (0.05 – 1 Hz; RMS = 0.3 Nm) to the manipulandum while subjects applied counter torques to keep the manipulandum aligned parallel to their sagittal plane. No visual feedback of arm position was provided during the torque perturbations so as to constrain sensory feedback to the proprioceptive path.

Subjects’ internal prediction of their visual and proprioceptive response delays *(T*_v_^*^, T_p_^*^) were estimated based on the timing and duration of corrective submovements in Tasks 1 & 2 respectively. Computationally, submovements have been associated with a discretization of corrective movements that minimize movement error and energy expenditure during closed-loop sensorimotor control [42–45]. Analysis of corrective submovements can also provide an estimate of the visual response delay [46] along with other neurocomputational processes associated with movement planning and execution during a corrective movement.

To account for passive dynamics of the arm in the measured torque response during the second task, an additional five “control” trials (30 sec. each) were collected during which subjects were instructed not to apply corrective torques (i.e. subjects were instructed to maintain the same posture and level of stiffness as in the other tasks, but to not otherwise interfere with the task). During these trials, a high frequency torque perturbation was applied to the arm (0-30Hz, first-order zero-phase Butterworth filter with 1Hz cutoff). The contribution of the passive mechanical impedance of the arm to the measured torque was estimated from the least-squares linear regression between the measured and applied torques during the passive trials (R^2^ > 0.75). The contribution of the passive mechanical impedance of the arm was then subtracted from the measured torque to estimate subjects’ voluntary corrective torque during proprioceptive task trials.

##### Task 3:

**Pursuit tracking of step torque** Signal-dependent motor variability (“motor noise”), was assessed using an isometric task adapted from Jones et al. [37], which measured joint torque variability as a function of average joint torque. During the task, the manipulandum position was fixed parallel to the subject’s sagittal plane while subjects produced several isometric torque contractions. Displacement of the cursor from the center of the screen scaled in proportion to the torque applied to the manipulandum. The subject was required to place the cursor on one of five targets (desired elbow joint torques of 4, 6, 8, 10, and 12 Nm flexion) by applying the appropriate isometric contraction. Five trials were collected at each of the five torque levels (25 trials total). During each eight-second trial, visual feedback of the target and cursor was shown for the first three seconds. Visual feedback was then removed, while subjects attempted to maintain the specified torque level for remaining five seconds.

##### Compensatory tracking with high-frequency perturbations

 A high-frequency compensatory tracking task was used to characterize the remaining elements of the sensory feedback control model including the controller gains (*K*_*d*_* , K*_*pr*_* , K*_*i*_), visual and proprioceptive feedback gains (*K*_*v*_* , K*_*p*_), and arm dynamics (*J, B, K*) together with their internal estimates (*K*_v_^*^, K_p_^*^, J^*^, B^*^, K^*^), vis-à-vis the forward model. During the task, high frequency, continuous, pseudorandom displacements (0–10 Hz, RMS = 20° visual angle, first-order zero-phase Butterworth filter with 1 Hz cutoff) were applied to the cursor. Subjects were instructed to make corrective movements as quickly and accurately as possible so as to maintain the cursor on a central stationary target. Ten 32-second trials were obtained for each subject.

##### Task 5:

**Pursuit tracking of step displacements** A step displacement task was used to characterize the functional impact of subjects’ deficits during a reach and hold task and compare target capture movements invoked by the subjects to those predicted by the sensory feedback control model of Figure 1. Subjects performed ten trials of the task. Each 10-second trial started with the target and cursor located at the same screen position. After a one second delay, the target was randomly displaced to the left or right by a randomly selected distance ranging ±24.4 cm along the horizontal midline of the display (corresponding to ±11.5 degrees of visual angle). Subjects were instructed to center the cursor on the target as quickly and accurately as possible and to maintain the cursor position until the end of the trial.

### Data analysis

#### Intention tremor frequency and power

Tremor frequency and power were quantified using each subject’s performance during the spiral drawing task. For each spiral trace, we performed a least-squared-error linear regression of the pen-tip trace angle vs. radial distance from the spiral’s center (Matlab command: polyfit) to remove the linear increase in angular position associated with the spiral. The best-fit regression was subtracted from the pen-tip data to obtain the variation in the subject’s movement about the spiral trajectory. The power spectrum of the residual pen-tip data was calculated for each trace and the best-fit (1/f) frequency spectrum was subtracted to account for low-frequency (<1 Hz) drift and to isolate the spectral power due to tremor. Tremor frequency was defined as the frequency that contained the maximum power in each trial; frequencies were averaged across trials to estimate the average tremor frequency. Tremor amplitude was defined as the maximum power in the tremor frequency range (2–6 Hz) for the trial performed at each subject’s fastest speed; this frequency range was chosen from the distribution of upper limb tremor frequencies associated with kinetic tremor [2].

#### Model parameter estimation

##### Sensory delays (Tasks 1 & 2

 We used cross correlation analysis to estimate delays in the visual and proprioceptive feedback-driven responses to band-limited low-frequency perturbations applied in the two low-frequency compensatory tracking tasks (Tasks 1 & 2). The visual response delay, *T*_*v*_, was estimated as the trial-wise average of the temporal offset (lag) between the perturbations in cursor position applied in Task 1 and the subject’s corrective responses measured by the robot’s handle position. The proprioceptive response delay was obtained by correlating the subject’s voluntary corrective torque response in Task 2 with the applied torque perturbations. The proprioceptive response delay, *T*_*p*_, was estimated as the trial-wise average of the temporal lag between the continuous torque perturbations applied to the arm in Task 2 and the subject’s voluntary corrective responses.

We estimated each subject’s internal prediction of their visual and proprioceptive response delays *(T*_v_^*^, T_p_^*^) using the average interval between successive corrective submovements measured in Tasks 1 & 2, respectively. Submovement intervals were defined as the times between zero-crossings of the elbow angular velocity. We accounted for event detection failures in each task (i.e. overestimation of submovement intervals) using a Gaussian mixture model fit to the distribution of submovement intervals across trials. In each task, the means (and variances) of the component Gaussians in the mixture model were constrained to be integer multiples of the primary (i.e. shortest) interval, thus reflecting a doubling and tripling of interval durations (and variability in their estimates). The internal estimate of the visual and proprioceptive response delays were taken as the means of the primary distributions of submovements in Tasks 1 & 2, respectively.

##### Signal-dependent motor noise (Task 3)

 The gain of the multiplicative (signal-dependent) motor noise, α, was estimated using the torques measured during Task 3, which involved pursuit tracking of step torque targets. For each target torque level, the mean and variance in the applied torque was measured during the last five seconds of each trial (i.e. after visual feedback was removed). The gain of the multiplicative noise ***α*** was estimated as the slope of the linear regression between the mean and the variance of the trial-averaged torque as a function of target torque level.

##### **Frequency response analysis (Task 4)**

 For each subject, we estimated the remaining model parameters (*K*_*d*_* , K*_*pr*_* , K*_*i*_* , K*_*v*_* , K*_*p*_ , *J, B, K*), together with the internal estimates (*K*_v_^*^, K_p_^*^, J^*^, B^*^, K^*^) using a two-stage frequency response analysis, which related the experimentally-imposed cursor and torque perturbations to compensatory changes in arm position. During the analysis, each subject’s sensory delays and motor noise parameters were held constant at values derived during the analysis of data from the first three tasks. The remaining model parameters were fit to each subject’s responses in the frequency domain using the simplex method (Matlab: fminsearch).

In the first stage of the analysis, the second-order model of musculoskeletal dynamics (Eq. 2) was fit to the magnitude of the frequency response function (FRF) relating the subject’s arm position to the applied torque. To reduce measurement noise prior to the model fit, FRFs were computed for all pair-wise combinations of trials as the ratio of the trial-wise differences between the applied torque (τ) and measured arm position (*θ*_*a*_) (see Appendix), and then averaged:

$$FRF\left(s\right)=\frac{1}{M}\sum_{i=1}^{N}\sum_{\begin{array}{c} j=2\\ j>i \end{array}}^{N}\frac{\theta_{a_{i}}\left(s\right)-\theta_{a_{j}}\left(s\right)}{\tau_{i}\left(s\right)-\tau_{j}\left(s\right)}\text{.}$$

where N is the number of trials and M is the total number of trial-wise pairs.

In the second stage of the analysis, the remaining model parameters were estimated from the closed-loop transfer function relating the applied visual perturbation (*D*_*ext*_) and measured arm position (*θ*_*a*_):

3 $$\theta_{a}\left(s\right)=-\left[\frac{K_{v}C\left(s\right)P\left(s\right)e^{-T_{v}s}}{1+C\left(s\right)P^{*}\left(s\right)-C\left(s\right)P^{*}\left(s\right)*\left(K_{v}^{*}e^{-T_{v}^{*}s}+K_{p}^{*}e^{-T_{p}^{*}s}\right)+C\left(s\right)P\left(s\right)\left(K_{v}e^{-T_{v}s}+K_{p}e^{-T_{p}s}\right)}\right]D_{ext}\left(s\right)$$

where,

4 $$P^{*}\left(s\right)=\frac{1}{J^{*}s^{2}+B^{*}s+K^{*}}$$

characterizes the forward model prediction of arm kinematics. The closed-loop model (Eq. 3) was fit to a separate FRF formed from the ratio of trial-wise differences between the applied perturbation (*D*_*ext*_) and measured arm position (*θ*_*a*_) computed for all pair-wise combinations of trials (See Appendix). Visual and proprioceptive feedback gains (K_v_^*^, K_p_^*^) were assigned using the subject’s fitted sensory gains (K_v_, K_p_). Motor noise (α), visual $\left(T_{v},T_{v}^{*}\right)$ and proprioceptive $\left(T_{p},T_{p}^{*}\right)$ response delays were fixed at the mean values estimated from tasks 1–3.

Phase data was excluded from the model fit due to the noise in FRF phase estimates, particularly at higher (>2 Hz) frequencies where the power of both the input signal and the subject response were attenuated. For frequencies below two hertz, the model and FRF phase profiles were driven primarily by the visual delay in the system, which was more accurately estimated using the cross-correlation between the applied perturbations and the subject’s corrective response.

We performed bootstrap analysis for each stage of analysis to quantify uncertainty in our estimates of the FRF and to quantify sensitivity of parameter estimates to measurement noise and model initial conditions. For each bootstrap, 10,000 model fits were performed (with random sampling of the initial conditions for each parameter and of the FRF data points included in each fit): Initial conditions for each parameter were selected from a uniform distribution spanning one order of magnitude centered on nominal values estimated across subjects in a previous analysis [33]; Three hundred data points were selected randomly with replacement across the three-decade range of the FRF. Model fits that did not converge to a solution within 400 iterations due to poor initial parameter estimates (~10% of cases) were discarded from subsequent analysis. For the remaining fits, the mean and standard deviation of the fitted parameters were used to estimate the nominal best-fit value and magnitude of uncertainty in the model parameters. For the second-stage bootstrap, plant parameter triplets (J, B and K) were randomly sampled from the first-stage FRF analysis to propagate the accumulated error across sequential model fits. During the second stage fits, these triplets were held constant.

For completeness, model and FRF phases for each subject were compared post-hoc using the best-fit model parameters to the FRF magnitude. Due to the noise in FRF phase estimates at higher frequencies, phase profiles could not be reliably “unwrapped” using a one sample unwrapping procedure. Instead, a multi-sample unwrapping procedure was implemented using a linear regression of phase estimates across the preceding 20 frequency samples to generate a 95% confidence interval around the location of the next “unwrapped” phase value. The FRF phase was then unwrapped by adding or subtracting multiples of 2**π** until the estimate fell within the confidence interval. In cases were the phase estimate could be unwrapped to two or more locations within the confidence interval, the median was chosen. Uncertainty in the phase profile resulting from the interaction between the unwrapping procedure and the occurrence of multiple phase estimates for confidence intervals exceeding 2**π** were quantified using a bootstrap analysis. During the bootstrap analysis, the unwrapping procedure was applied to the FRF phase estimates 1000 times, randomly sampling the phase at each frequency containing two or more unwrapped phase estimates within the confidence interval. The 95% confidence interval associated with the unwrapped phase profile was defined at each frequency from the distribution of samples obtained from the bootstrap analysis.

##### **Pursuit tracking of step target displacements (Task 5)**

 We evaluated the ability of each subject’s best-fit model to characterize sensory feedback control in a separate task that required pursuit tracking in response to step displacements (task 5). For each subject, the trial-wise measures of target acquisition time and mean squared endpoint error were calculated. Target acquisition time was calculated as the time required for the subject to move within two degrees of the target. Endpoint error was calculated as the mean-square error (MSE) from the moment of target acquisition to the end of the trial. Target acquisition time and endpoint error were then compared with those of the best fit model to determine the extent to which subject performance was constrained by limitations of sensory feedback control as identified by the model of Figure 1.

##### **Statistical testing**

 Healthy subjects were matched to patients by age and gender to control for differences in movement control due to factors unrelated to MS. Group differences in the measurements of visual and proprioceptive response delays, submovement intervals, motor noise, and best-fit estimates of the model parameters were tested for statistical significance using a paired, two-sample t-test. Within-subject comparisons of the parameters characterizing internal (predicted) and actual passive limb dynamics were tested for statistical significance using the paired samples z-score of the bootstrap distributions to evaluate the distribution difference from zero (i.e., no difference between distribution means). Pearson’s correlation coefficient was applied across participants with MS to identify significant linear relationships (p < 0.05) between all combinations of best-fit model parameters and the quantitative clinical assessments of movement performance (e.g. 9HPT, TAS). Post-hoc analysis of the relationship between spiral tremor power and the difference between the internal (predicted) and actual passive limb dynamics was characterized empirically using a least-squares fit to a saturating exponential function of the form

$$\Delta \left(p\right)=C*\left(1-e^{-r*x}\right)$$

where C is a scaling factor, r is a constant, and x is tremor power.

## Results

### Tremor frequency and power

**Figure 3:**
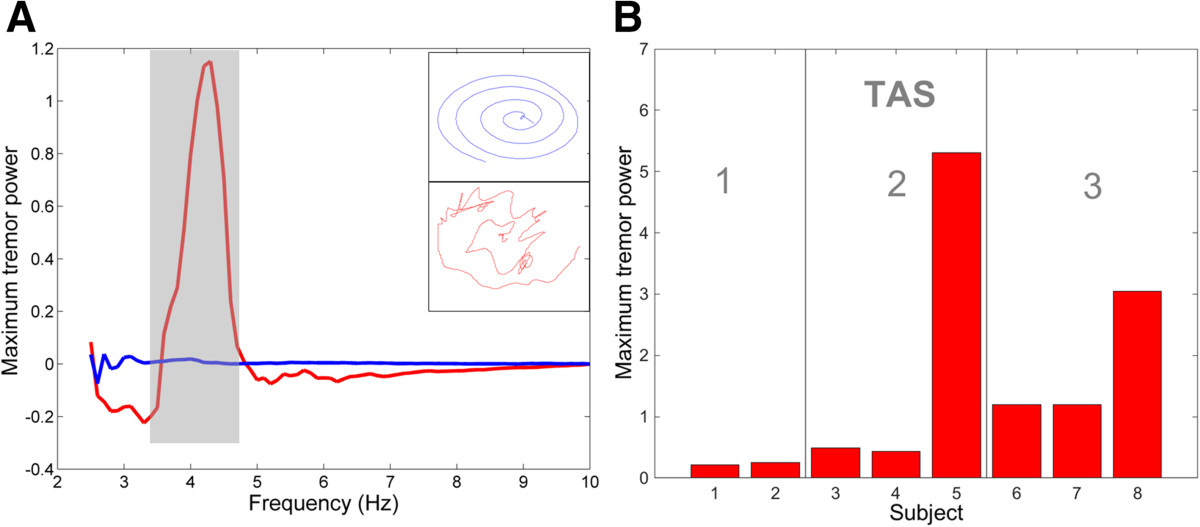
**Tremor assessment using the spiral drawing task. (A)** Power spectra (with low-frequency drift removed) and sample spiral drawings (inset) for Subject 6 with MS (red; TAS = 3) and an age-matched control subject (blue). The shaded area highlights the range of frequencies associated with the subject’s tremor. **(B)** Maximum power within the 2–5 Hz frequency range for subjects with MS together with their corresponding tremor assessment score (TAS).

Figure 3A shows selected spiral traces (insets) and corresponding power spectra for a subject with MS (Subject 6; TAS = 3) and an age-matched control subject. In the spiral drawing task, tremor frequencies for subjects with MS ranged from 2.36-5.01 Hz (mean ± SD: 3.38 ± 0.91Hz). Within the 2–6 Hz range associated with tremor, maximum power increased with the speed of movement (data not shown) and ranged from 0.22-5.31 cm^2^-s (mean ± SD: 1.48 ± 1.82 cm^2^-s) across MS subjects for their fastest tracings (Figure 3B). The power in the 2–6 Hz band corresponded roughly with TAS, with subjects 5 (TAS = 2) and 8 (TAS = 3) exhibiting the worst tremor and subjects 1, 2 (TAS = 1), and 4 (TAS = 2), exhibiting the least tremor on the day of testing. Tremor power was significantly correlated with 9HPT score on the day of testing (r = 0.80; p = 0.006).

### Visual and proprioceptive response delays (Tasks 1 & 2)

**Figure 4:**
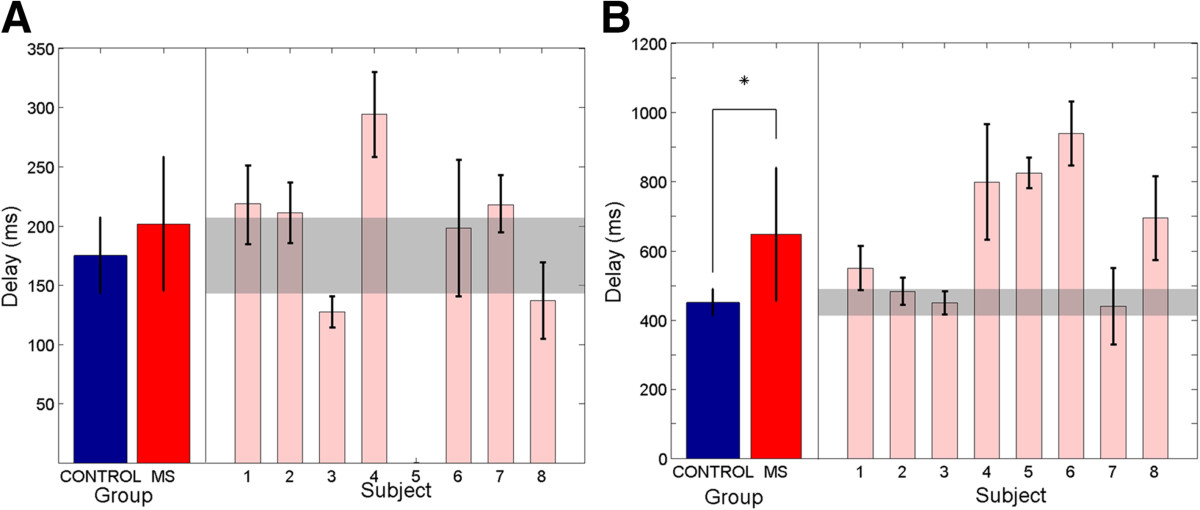
**Visual and proprioceptive response delays for control subjects (blue) and subjects with MS (red). (A)** Group visual response delays are shown on the left. Visual response delays for individual subjects with MS are shown on the right together. **(B)** Group proprioceptive response delays are shown on the left. Proprioceptive response delays for subjects with MS are shown individually on the right. Error bars denote ± SD for group and individual measures respectively. Shaded regions denote the corresponding ranges (±SD) for the control group.

Figure 4A and B show the average visual and proprioceptive response delays for individual subjects with MS and the corresponding range (±SD) for control subjects (shaded bands). The average visual response delay measured across subjects (Figure 4A-left), was significantly higher in subjects with MS (647.1 ± 192.3 ms), compared with control subjects (450.9 ± 38.2 ms) (t(7) = 2.63, p = .034). In contrast, the average proprioceptive response delay (Figure 4B-right) did not differ significantly between groups (MS: 201.7 ± 56.5 ms; Controls: 175.1 ± 31.9 ms) (t(6) = 1.39, p = 0.21). In four of the eight MS subjects with elevated TAS scores (subjects 4, 5, 6, and 8), visual response delays were >3σ above the range of control subjects. Across subjects, visual response delay times were not significantly correlated with either TAS or spiral tracing performance (p > 0.25), likely due to the “outlier effect” of subject 7 on the small population sample. Individual proprioceptive response delays for subjects with MS fell within the control group range - excepting subject 4, whose proprioceptive delay was >2σ from the control average.

**Figure 5:**
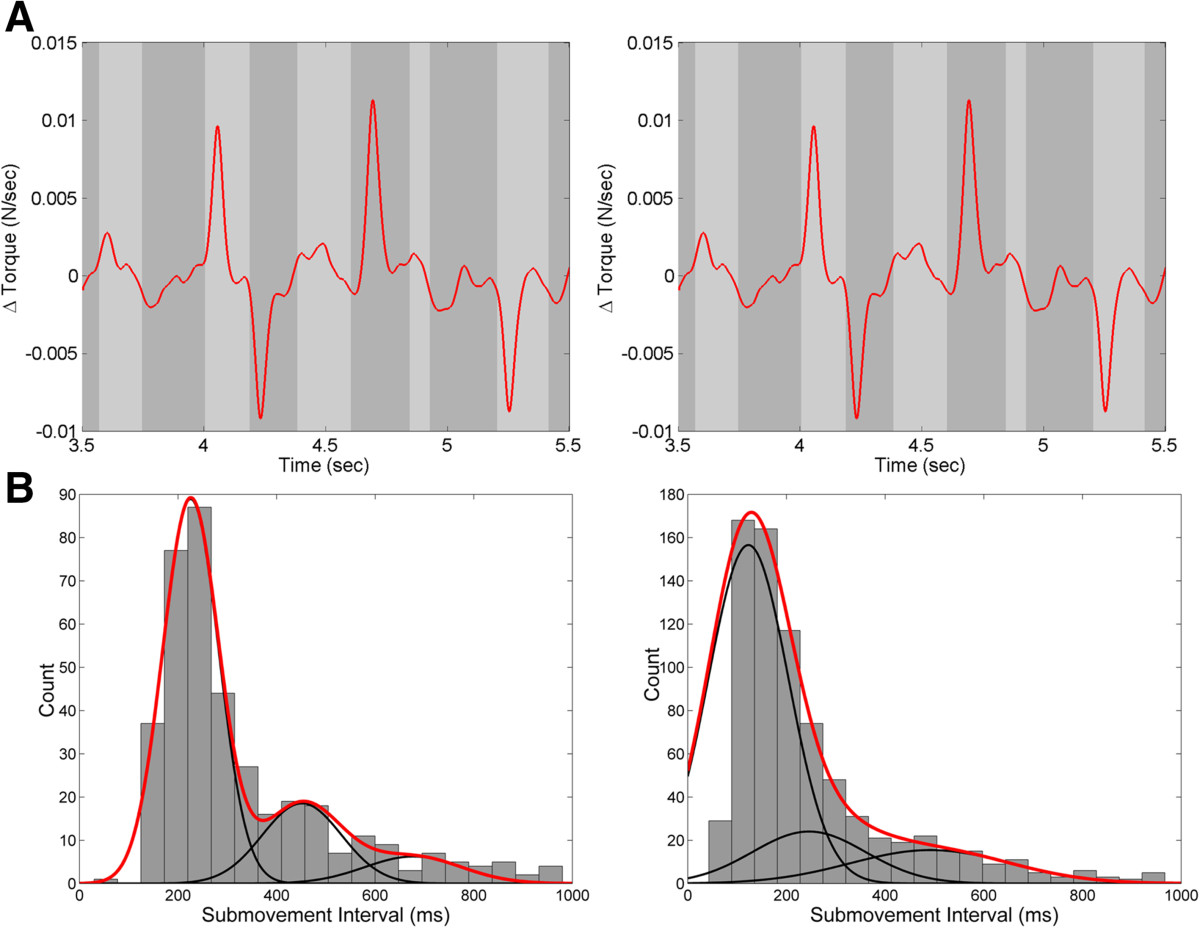
**Characterization of visual and proprioceptive submovement intervals. (A)** Movement velocity profiles used to calculate visual (left) and proprioceptive (right) submovment intervals for Subject 4 (MS, TAS = 2). Examples of individual submovements are highlighted (gray) **(B)** Distribution of submovement intervals across trials for vision (left) and proprioception (right) for a representative subject with MS (Subject 4). The submovement interval for each subject was characterized by the mean and standard deviation of the best-fit gaussian mixtures model (red line) formed from successive gaussian functions whose means and variances are constrained to be integer multiples of the primary distribution (black lines).

**Figure 6:**
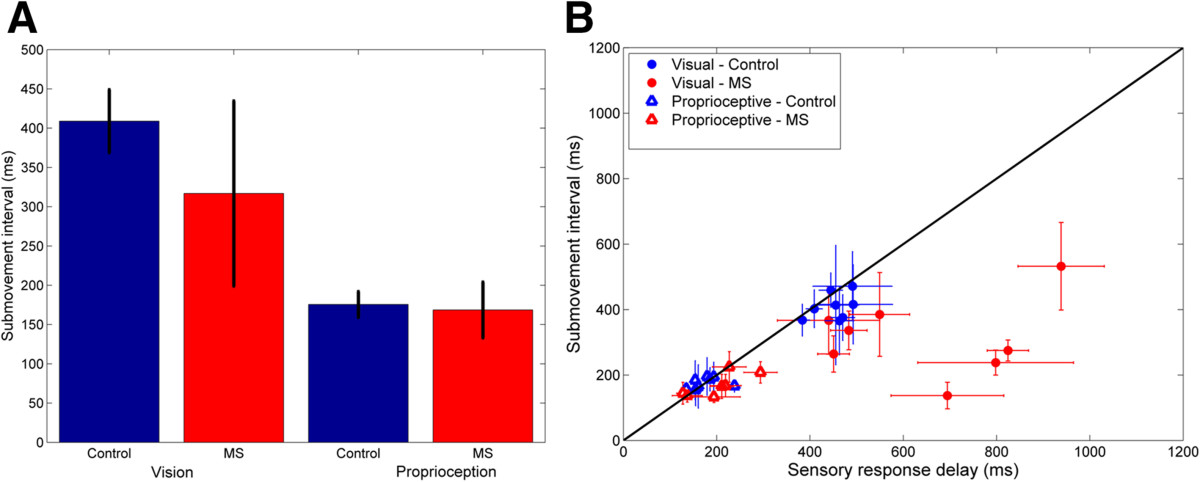
**Comparison of submovement intervals and task response delays. (A)** Group average internal visual response delay (±SD) for control subjects (blue) and subjects with MS (red). **(B)** Visual (filled circles) and proprioceptive (open triangles) response delays (±SD) as a function of submovment interval for control subjects (blue) and subjects with MS (red). The diagonal line (black) represents equivalency between response delay and submovement interval.

Figure 5 shows representative single-trial velocity profiles within trial and the distributions of visual and proprioceptive submovement intervals across trials for a representative subject with MS in Task 1 (left), which included visual perturbations and in Task 2 (right), which included physical perturbations. For all subjects, distributions were well fit by the Gaussian mixtures model (r^2^ > 0.70 p < 0.001 with 3 Gaussians) wherein the mean of each Gaussian was centered at an integer multiple of the interval associated with the primary distribution. For each subject, internal (predicted) visual and proprioceptive response delays were estimated as the mean submovement interval of the primary distribution.Figure 6A shows the average visual and proprioceptive submovement intervals across subjects. Proprioceptive submovement intervals did not differ significantly between the MS and age-matched control groups (t(6) = 1.88, p = 0.11). Visual submovement intervals tended to be shorter in subjects with MS compared to controls, however, the difference did not reach statistical significance (t(7) = -1.92, p = 0.097). Figure 6B compares the duration of visual and proprioceptive response delays for each participant with their corresponding submovement intervals. Proprioceptive submovement intervals and response delays were approximately equal for both control and MS subjects (t(9) < 1.6, p > 0.05). Similarly, visual response delays and submovement intervals did not differ for control subjects (t(9) < 1.4, p > 0.05). By contrast, four of the eight MS subjects exhibited a dramatic mismatch between their visual submovement interval and corresponding visual response delay. In these subjects, visual response delays increased markedly compared to control subjects, resulting in a significant group difference between visual response delay and visual submovement interval (t(7) = 2.55 p = 0.038).

### Motor noise (Task 3)

One subject with MS (Subject 5) was unable to complete the task due to time constraints. For the remaining subjects, the scaling of elbow torque variability with mean elbow torque showed no significant differences between groups (control subjects: 0.021 ± 0.010; subjects with MS: 0.025 ± 0.011; paired samples: t(6) = 0.72, p = 0.48).

### Frequency response analysis (Task 4)

**Figure 7:**
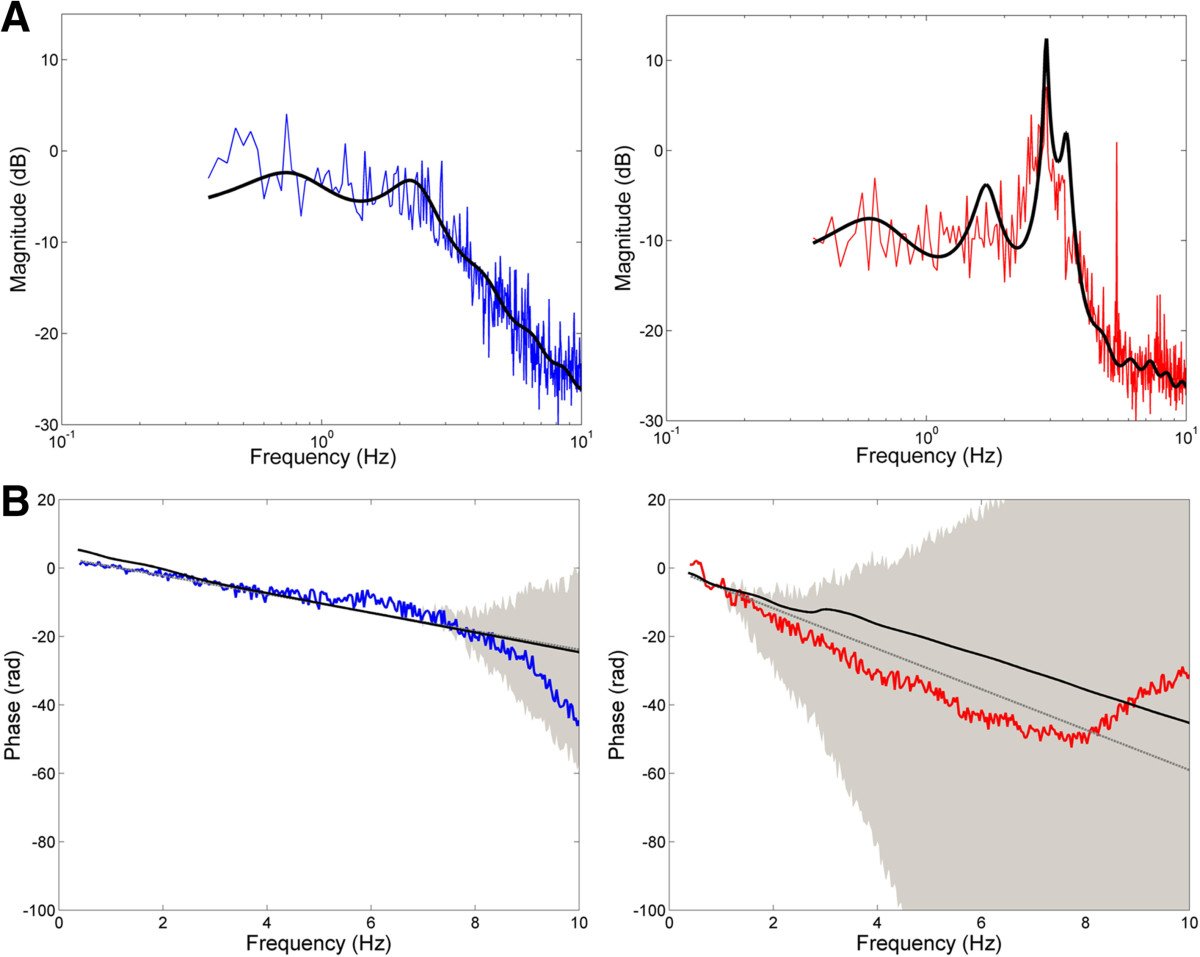
**Subject frequency response functions (FRFs) and model fits.** **(A)** Magnitude of the FRF (colored traces) relating applied cursor perturbation to corrective change in arm position for subject 4 with MS (TAS = 2; right) and the age-matched control subject (left). The best-fit model for each subject is denoted by the solid black line. **(B)** Phase of the FRF (colored traces) with 95% confidence intervals (grey shading) for subject 4 with MS (right) and age-matched control subject (left). The solid black line denotes the best-fit model to the subject’s magnitude FRF. The grey line denotes the phase profile associated with the subject’s visual delay.

The frequency response functions (and corresponding best-fit models) relating corrective changes in arm position to the perturbation of cursor position are shown in Figure 7 for subject 4 with MS (right) and the corresponding age-matched control (left). For subject 4, the empirical frequency response function and corresponding model fit both contain a marked resonance peak between 2–4 Hz, closely approximating the tremor frequency observed in the subject’s spiral tracing task (i.e., 2.4-5 Hz). The peak frequency identified in the compensatory tracking task was slightly lower than in the spiral tracing task, likely due to the additional inertia of the manipulandum handle and robot, which would act to reduce the resonant frequency of the combined arm + robot system. The magnitude of the FRFs for all subjects (control and MS) were well approximated by the model of Figure 1 (R^2^ > 0.80 in every case). The phase of the FRF was well approximated by the model until approximately 2Hz and 6Hz in the MS patients and control subjects respectively. Within this range, the phase profile was dominated by the phase lag associated with the visual delay (Figure 7B – gray line). At higher frequencies, phase estimates became too noisy to unwrap reliably, however, model responses fell within the 95% confidence interval of possible phase profiles unwrapped from the FRF phase.

Of the thirteen parameters estimated using the frequency responses analysis, significant differences between groups were observed only for the integral and derivative gains of the generic feedback controller. Subjects with MS exhibited higher integral gains than control subjects (6.86 ± 4.71 vs. 4.71 ± 2.39 Nm/deg-s; t(7) = -3.62, p < 0.01) and higher derivative gains than control subjects (8.3×10^-3^ ± 3.8 ×10^-3^ vs. 3.3 ×10^-3^ ± 1.8 ×10^-3^ Nm-s/deg; t(7) = -3.38, p < 0.05). In control subjects, the derivative gain was significantly correlated with integral gain, musculoskeletal viscosity, and musculoskeletal stiffness (r = 0.81, 0.71, and 0.75 respectively; p < 0.05). In subjects with MS, these correlations were absent; derivative gain was not correlated with actual (or predicted) musculoskeletal viscosity or stiffness and it was not correlated with tremor assessment score and tremor amplitude measured by the spiral tracing task (r < 0.50; p > 0.25). Instead, the best-fit derivative gain was significantly correlated with visual response delay in subjects with MS (r = 0.77; p = 0.024). This shift in coupling from the plant (in controls) to the visual delay (in subjects with MS) is interesting in light of the derivative gain’s traditional role in modulating the transient response of the system. This finding suggests the increased visual processing delay seen in MS may play a central role in causing subjects to alter the effective closed-loop dynamic response of the arm during goal-directed movement.

**Figure 8:**
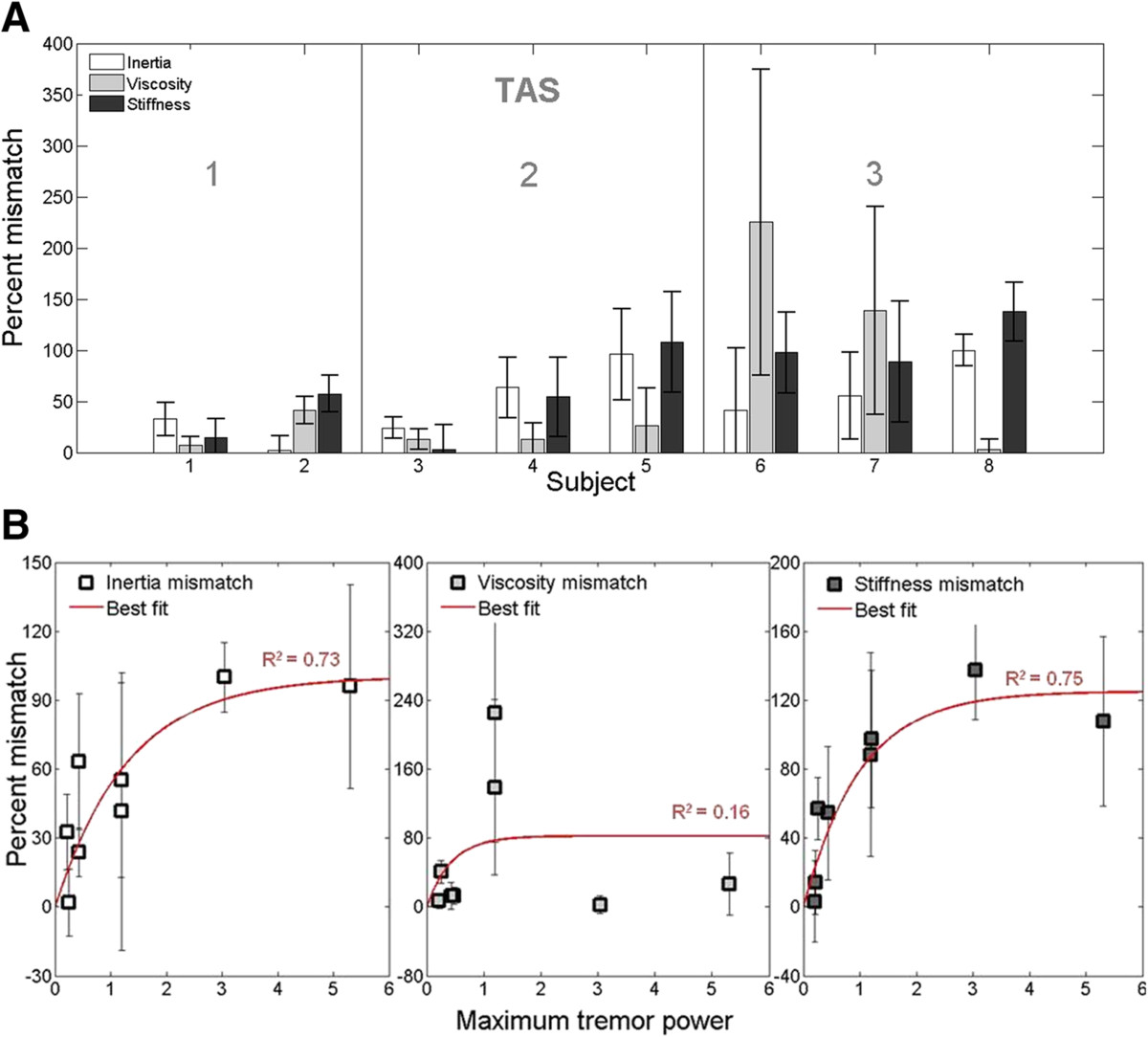
**Mismatch between predictive and actual limb dynamics.** **(A)** Percent mismatch between predictive versus actual estimates of passive joint dynamics (inertia, viscosity, stiffness) as a function of tremor severity (TAS score) in subjects with MS. The mismatch between actual and predicted limb dynamics increased with tremor assessment score. Error bars denote ± SD of the bootstrap distribution. **(B)** Percent mismatch between the parameters characterizing internal (predicted) and actual passive joint dynamics for subjects with MS (±SD), plotted against tremor power characterized using the spiral-tracing task. Percent mismatch increased as a saturating function of with tremor magnitude (red) for inertia and stiffness (R^2^ > 0.70; p < 0.01) but not for viscosity (R^2^ = 0.16; p = 0.32).

We next analyzed the best-fit sensory feedback control models from subjects with MS to identify systematic covariations between model parameters and clinical performance measures. We found that subjects with MS displayed a consistent mismatch between the model parameters characterizing predictive arm dynamics (Eq. 4) and the actual arm dynamics (Eq. 2). The degree of parameter mismatch - quantified by the mismatch magnitude normalized by the corresponding parameter value from the actual arm dynamics - varied systematically with tremor assessment score (TAS). Mismatches in all three dynamical parameters (*J*, *B* and *K*) increased with tremor severity, although mismatches in the effective viscosity were evident only in subjects with severe tremor (TAS = 3), (Figure 8A). By contrast, control subjects showed no mismatch between the parameters characterizing internal and actual passive joint dynamics (two-tailed Z < 1.9, p > .05 for each parameter).

Mismatches in inertia and stiffness also varied systematically with tremor power characterized using the spiral tracing task (Figure 8B); In both cases, the relationship was well approximated by a saturating exponential function (R^2^ > 0.73). By contrast, no systematic relationship was observed between mismatches in viscosity and tremor power (Figure 8B).

### Pursuit tracking of step target displacements

**Figure 9:**
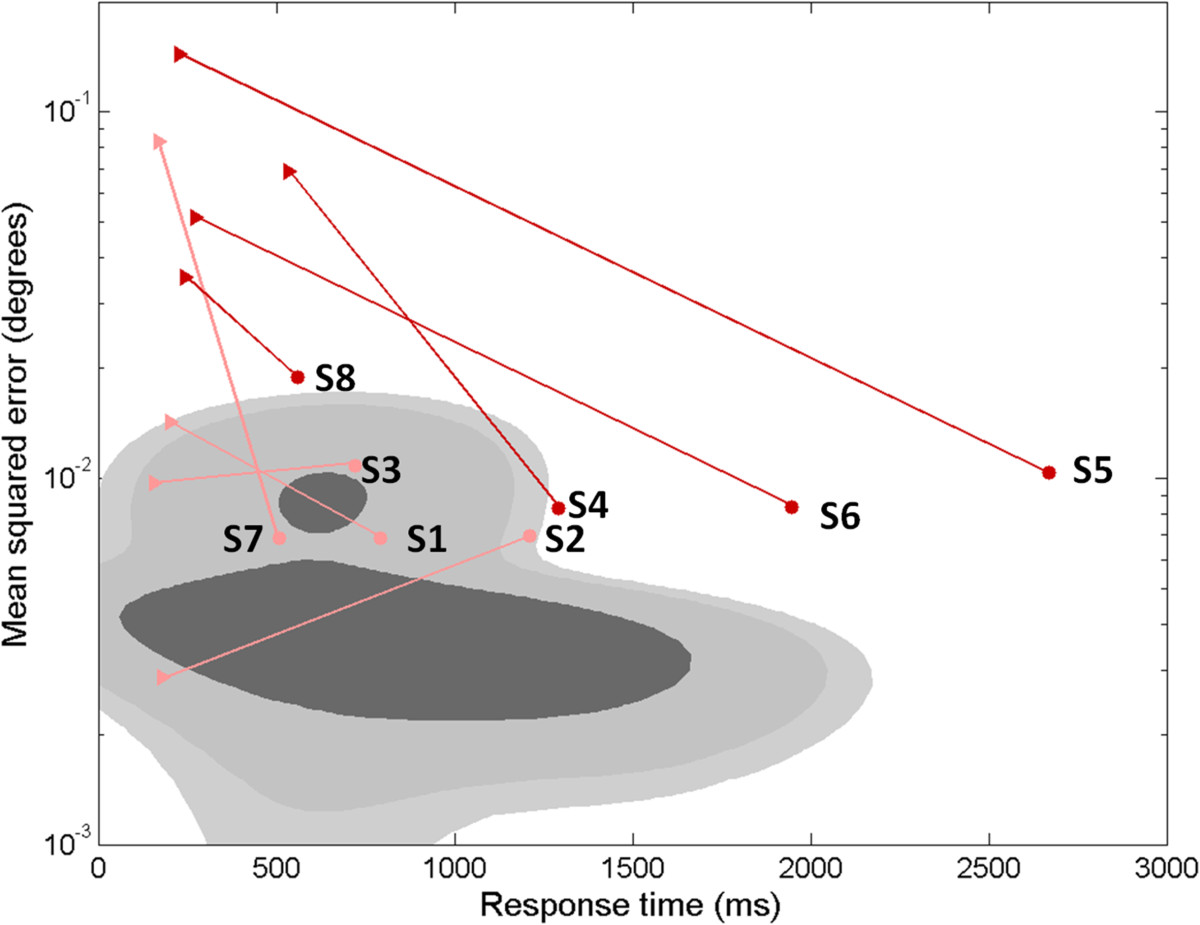
**Steady state error (degrees) vs. response time (ms) during a reach and hold task (step displacement).** Shaded regions (dark, medium, and light gray) denote the 50, 90, and 95% confidence intervals estimated from a mixture of Gaussians fit to control subjects’ response across all trials. For subjects with MS, trial-averaged response times and MSEs are shown individually for clarity (filled circles). Dark red symbols denote MS subjects with “high” (>3SD above the control mean) visual delays, and pink symbols denote MS subjects with “low” (<3SD) visual delays. The average best-fit model performance to the same trials is also shown for each subject (filled triangles). Four subjects (all with “low” visual delays) lie within the 95% CI for control subjects. Four subjects (all with “high” visual delays) lie outside the 95% CI for control subjects. In all cases, the best-fit sensory feedback control model for subjects with MS (filled triangles) reacted more quickly to a target perturbation than the subjects’ actual responses (filled circles).

We required subjects to perform a final tracking task to characterize the impact of sensory feedback control deficits on a reach and hold task similar to transporting a cup of water along a tabletop. The task also enabled us to compare movements generated by the subjects to those predicted by the sensory feedback control model of Figure 1. Subject and model performance were examined using measures of target acquisition time from the onset of the step displacement and steady-state endpoint error following the displacement. Control subjects’ performance tended to cluster into one of two general task strategies characterized by either larger endpoint errors and faster response times or smaller endpoint errors and slower (and more variable) response times (Figure 9; note the two distinct peaks in the bivariate distribution of control subjects’ performance represented by the dark shading). For responses emphasizing speed of movement (higher error, lower response time), 95% of trials took less than 1200 ms to reach the target (Figure 9, top shaded distribution) and resulted in an endpoint MSE’s up to 0.02 degrees^2^. For responses emphasizing endpoint accuracy, 95% of trials were completed within 2000 ms with endpoint MSE’s less that 0.008 degrees^2^ (Figure 9, bottom shaded distribution).Subjects with MS exhibited similar trends in step-tracking performance, with the exception that the four subjects with high visual delays (Figure 9, dark red circles) exhibited performances that fell outside the 95% confidence interval bounds of the bivariate distribution of the response times and endpoint MSEs exhibited by control subjects. The subjects with high visual delays all had high TAS and high tremor power. Three of the four subjects (S4, S5, and S6) had significantly higher response times when performing the step-tracking task. Endpoint MSE was also increased, falling within the range of control responses emphasizing speed over accuracy. The fourth subject (S8) showed the reverse pattern with an increase in endpoint MSE but no apparent increase in response time. For all subjects with MS, the corresponding performance of the best-fit model, averaged across trials, is shown for comparison (Figure 9, triangles). In all cases, model-predictions underestimated actual response times and in all but two cases, model-predictions over-estimated actual terminal mean-squared errors.

### Functional impact of mismatch between actual and predictive limb dynamics

**Figure 10:**
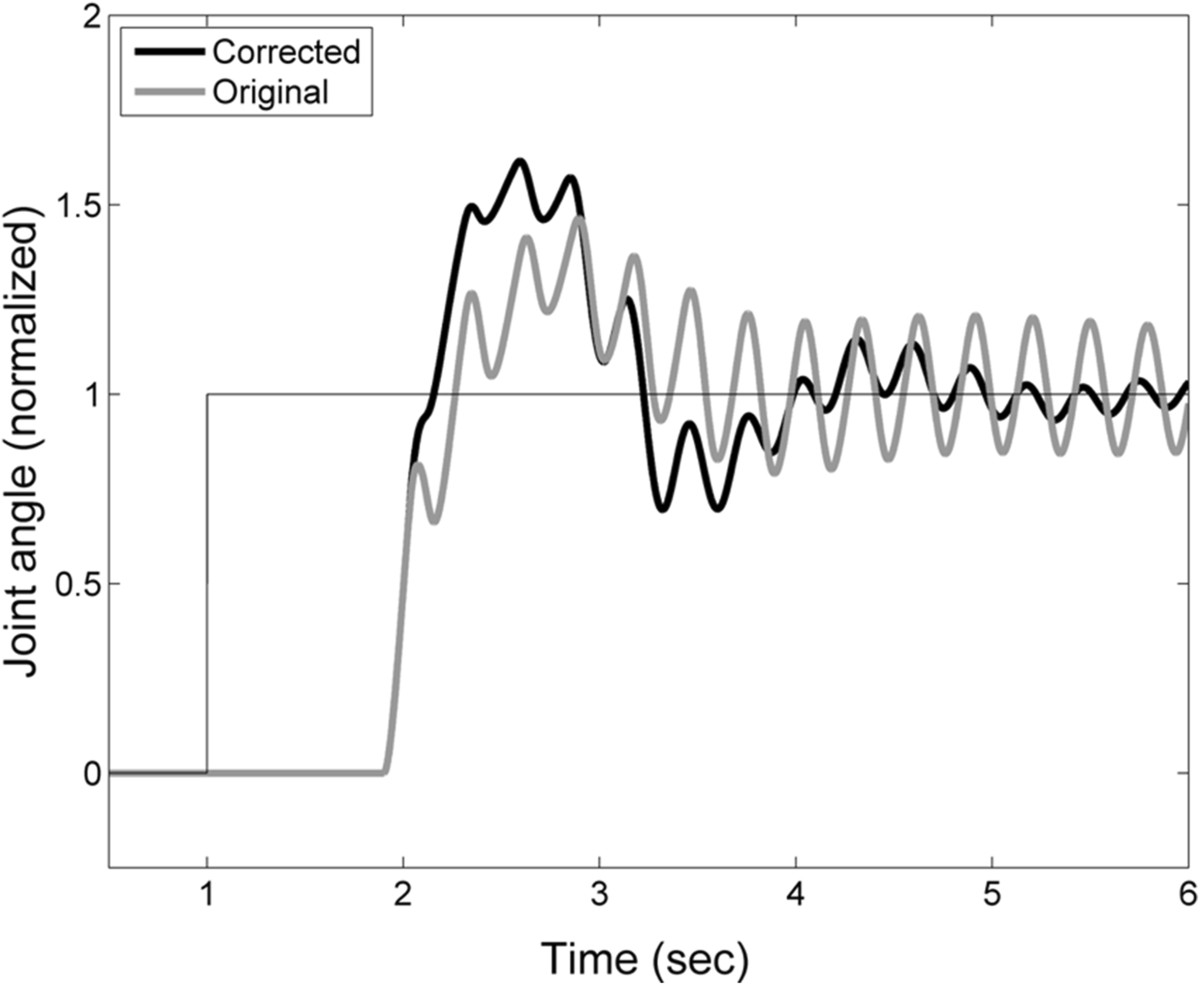
**Step response of the best-fit sensory feedback control model for subject 4 with moderate tremor (TAS = 2).** Response for the best-fit model containing a mismatch between the actual and expected elbow kinematics (black line) and for a model in which the actual and expected kinematics are matched (gray line).

We examined the functional consequence of the mismatch between actual and predictive arm dynamics in a subsequent, post-hoc simulation analysis. For each MS subject we performed two forward dynamic simulations that characterized the model’s performance on the step displacement task using (a) the best-fit model parameters, including mismatches between actual and predictive limb dynamics; and (b) “corrected” model parameters wherein the predictive limb dynamics of the forward model were forced to match the actual limb dynamical parameters. Figure 10 shows representative results for a subject with MS with moderate tremor (TAS = 2). Note how the mismatch in limb dynamics actually decreased the time to target acquisition and resulted in lower endpoint error.

## Discussion

We used a multisensory model of sensory feedback control to individually characterize sources of sensorimotor dysfunction in subjects with MS performing a series of goal-directed stabilization and movement tasks about the elbow. In contrast to the initial supposition that MS might impact sensory feedback control uniquely in each subject, the results suggest that upper extremity tremor and dysmetria may result from systematic changes in sensory feedback control. Specifically, subjects with moderate to severe tremor (TAS ≥ 2) exhibited increased visual response delays relative to normal control subjects. They also exhibited systematic mismatches between predictions of arm dynamics (vis-à-vis the forward model) and actual arm dynamics which were not present in normal control subjects; the degree of mismatch in subjects with MS correlated with tremor signal power measured in our spiral tracing task. We also observed group-wise differences in the integral and derivative gains of a generic model of the neural feedback controller. Whereas the controller gain parameters covaried with the dynamic properties (i.e., apparent viscosity and stiffness) of the musculoskeletal system in normal control subjects, the derivative gain parameter in subjects with MS correlated instead with the visual delay. A comparison of actual and simulated responses to step changes in desired performance suggests that the apparent mismatch between subject predictions of arm dynamics and actual arm dynamics may actually serve to improve response times in subjects with MS, despite their long visual delays. Taken together, our results suggest that tremor and dysmetria in MS may be caused by a combination of two factors: an inability of the brain to adequately adapt to increases in the time required to process visual information related to movement and by compensatory – but maladaptative – errors in predictions of arm dynamics.

An increased visual delay such as the one observed here is consistent with reductions in the conduction speed of action potentials due to disease-induced demyelination in MS [47] and it agrees well with the increased time required by MS subjects to perceive visual information and perform visually-guided tasks [47–49]. Proprioceptive conduction time in the lower extremities has also been shown to increase in MS [50], although we did not find a corresponding increase in proprioceptive response delay for the upper extremity. This may be due to the longer path length in the spinal cord for the transmission of motor control signals to the lower extremities.

Interestingly, the increased visual response delay in subjects with MS was not accompanied by an increase in the latency of submovements (i.e. their submovement interval). Submovements have been used previously to study impairments in movement control [51, 52]. Current theories of intermittent control during goal-directed movement associate individual submovements with discretization of sensorimotor control, such that each submovement represents a complete “primitive” movement profile comprised of movement planning, movement execution and sensory feedback phases [42–45]. For the purpose of characterizing feedback control in MS, we have assumed that the combined time delays associated with these three submovement phases form the basis of the expected response delays characterized by the model (Figure 1). Correspondingly, the submovement intervals measured experimentally in response to corrective movements mediated by visual or proprioceptive motion cues (Exp. 1a and 1b respectively) reflect internal estimates of the open-loop sensory processing delays. This interpretation is supported by the consistent match in control subjects between visual and proprioceptive response delays and the measured submovement intervals (Figure 6B).

In subjects with MS, submovement interval and visual response delay differed significantly in four of the eight subjects, suggesting that they failed to adjust (or were unable to adjust) their expectations of visual processing delays to compensate for the full increase in visual processing time resulting from the disease. A previous study by Miall and Jackson has demonstrated that it is possible to adapt to increases in extrinsic feedback delays [46]. However, the visual delays seen here in subjects with MS were markedly larger than those that Miall and Jackson used to adapt their neurologically intact subjects (<300 ms). Moreover, the delays experienced by MS subjects reflect intrinsic, rather than extrinsic sources. It is possible that intrinsic sources of delay may not engage adaptive mechanisms that respond to task-specific changes in the environment (cf. [53]).

Although continuous control models, such as the one used here, make simplifying assumptions that neglect the impact of intermittent feedforward control actions, continuous control models have been shown to accurately predict human performance in a variety of single joint motor tasks that minimize the predictability of environmental or target perturbations [30–33]. Additional simplifications of our model include the use of a 1-D task to characterize movement control and the use of a second-order musculoskeletal plant model. These simplifications were made because the plant model of the arm becomes much more complicated with the inclusion of additional joints or by including higher-order models of muscle activation contraction dynamics [54]. We believe these simplifications are justified because the bandwidth limitations of the plant are dominated by the effects of the arm’s inertia and mechanical viscoelasticity rather than by low-pass filter properties of the activation/contraction dynamics - at least in quasi-isometric conditions such as the stabilization tasks studied here.

For subjects with MS, the pattern of mismatch in the limb dynamics (stiffness and inertia) co-varied with tremor assessment score and tremor power calculated from the spiral-tracing task (Figure 8). This was despite marked differences in task design; the model was characterized using single-joint compensatory tracking movements with the arm supported against gravity whereas the clinical assessments and spiral tracing required the subject to generate motion at multiple joints without arm support. Correspondence in the results of these disparate tasks is to be expected; a disease-related increase in visual processing time is expected to impact motor performance in any visuomotor task. The simulation results of Figure 10 suggest that the observed mismatch between internal estimates of plant dynamics and actual plant dynamics may actually help subjects with moderate tremor reduce steady-state movement error despite an inability to compensate for long visual delays. This form of compensation would not be unreasonable, particularly for adaptive mechanisms in the brain that seek to minimize discrepancy between the predicted and realized sensory consequences of actions (cf. [55]). Uncompensated increases in visual delay would yield lagged perceptions of arm position, compromising limb state estimation [20]. Considering that a delay in the limb’s response to descending motor commands also occurs when the hand grasps an object that is heavier than expected, an uncompensated lag in the visual perception of limb motion could be misconstrued as an unexpected increase in limb inertia. Therefore, increasing the internal estimate of limb inertia (Figure 10) could, within narrow limits, partially compensate the functional impact of inaccurate predictions of sensory delay. Beyond those limits, changes in the estimated limb dynamics could lead to increased joint torque production (intended to overcome an environmental load that is not in fact present) and inappropriate compensatory responses to the perceived error. This notion is consistent with the suggestion that intention tremor in MS is due, in part, to inaccurate voluntary corrections to errors in position [48]. From a neurological standpoint, cerebellar damage, which has been linked with tremor in previous studies ([16, 17, 56, 57]), could degrade pathways necessary for effective sensorimotor adaptations, causing inappropriate compensatory responses to become more likely, and exacerbate tremor severity.During the reach-and-hold task, subjects with MS tended to move more slowly than the control subjects. They also moved more slowly than the performance predicted by best-fit models of Figure 1. These results are consistent with a favoring of accuracy over speed in the pursuit tracking of step changes in target location and may reflect a strategic choice by subjects to minimize endpoint errors associated with delay and kinematic mismatches. This bias toward accurate (rather than fast) movements is not surprising since in many daily activities (e.g. eating, dressing) it is more important to bring the hand accurately to a desired spatial location than to do so with speed.

Our results suggest a possible reinterpretation of results of prior studies seeking to reduce tremor in MS. Tasks which force subjects to adapt to novel force fields or to perturbations [27–29] could allow subjects to “reset” maladaptive models and form a new model that is better able to compensate for long visual delays. Our results also suggest novel rehabilitative strategies for reducing intention tremor in subjects with MS. We envision at least two possibilities: one approach would require subjects to hold the handle of a rehabilitation robot while making goal directed movements within a simple virtual-reality environment. As training progresses, subjects would be required to adapt to slowly-increasing visuomotor delays while the robot would simulate mechanical loads that vary unpredictably from trial to trial, thus discouraging compensatory mal-adaptation of musculoskeletal property estimates. We speculate that providing practice in compensating for visuomotor delays while discouraging adaptation of limb dynamics will favor appropriate adaptive compensations for physiological visual processing delays, thereby mitigating tremor.

A second approach centers on the idea that the brain’s effort to minimize performance error hinders the ability to adapt to changes in the physiological visual delay. That is, we speculate the presence of a non-monotonic relationship between performance error and increases in predictive delay such that small increases in predictive delay would lead to increased errors, while large changes in expected delay could lead to optimal performance. This non-linear relationship may preclude the inherent adaptive mechanisms from matching the predictive delay to the true physiological delay. Rehabilitation under this approach would involve using the feedback control model (Figure 1) to identify and tailor the visual feedback to gradually shift the minimum performance error to the actual visual delay [58].

## Conclusion

The preliminary findings presented here demonstrate that systems identification techniques provide an informative framework for investigating how neuromotor disease affects motor control and the neuromotor causes of motor disability. Specifically, we have done so by examining deficits in the neural processes underlying upper extremity motor dysfunction in a small cohort of individuals with clinical diagnoses of Multiple Sclerosis. We found evidence that tremor and dysmetria may be caused by an inability of the brain to adequately adapt to increases in the time required to process visual information related to movement as well as by compensatory mal-adaptations of internal estimates of arm dynamics. Future studies should seek to confirm the findings reported here with a larger cohort of individuals with MS. Subsequent studies could then seek effective ways to reduce intention tremor by identifying strategies that mitigate motor instability due to slowed visual processing caused by MS.

## Appendix 1. Subtraction Analysis

A subtraction analysis was used to reduce the impact of noise on the estimate of the subjects’ frequency response function (FRF). For each trial, the relationship between the input to the sensorimotor control system and joint angle output can be expressed as:

$$\theta_{a}\left(s\right)=H\left(s\right)*X\left(s\right)+N\left(s\right)$$

where X(s) is the power spectrum of the input – either the torque or external perturbation – and N(s) is the power spectrum of all noise sources combined. H(s) is the transfer function relating the input X(s) to the output θ_a_(s). The sum of noise sources n(t) is assumed to be zero mean and characterized by a nominal spectrum N(s). In the frequency domain, the addition of noise results in a frequency dependent offset from the “true” FRF. This offset can be characterized as a random variable N_i_(s) with variance σ_n_^2^(s), whose mean corresponds to the average noise spectrum. To eliminate this offset, individual estimates of the FRF were obtained by pair-wise subtraction of the trial-wise input and output spectra. For a pair of trials,

$$\begin{array}{c} \theta_{a_{1}}\left(s\right)=H\left(s\right)*X_{1}\left(s\right)+N_{1}\left(s\right)\\ \theta_{a_{2}}\left(s\right)=H\left(s\right)*X_{2}\left(s\right)+N_{2}\left(s\right) \end{array}$$

subtraction yields

$$\theta_{a_{1}}\left(s\right)-\theta_{a_{2}}\left(s\right)=H\left(s\right)*\left(X_{1}\left(s\right)-X_{2}\left(s\right)\right)+\left(N_{1}\left(s\right)-N_{2}\left(s\right)\right)$$

so that the nominal noise spectrum is removed and the variance is now centered around 0. Rearranging this equation, we get:

$$H\left(s\right)=\frac{\theta_{a_{1}}\left(s\right)-\theta_{a_{2}}\left(s\right)}{X_{1}\left(s\right)-X_{2}\left(s\right)}+\frac{N_{2}\left(s\right)-N_{1}\left(s\right)}{X_{1}\left(s\right)-X_{2}\left(s\right)}$$

where the first term characterizes the difference FRF of the system and second reflects the contribution due to noise. The frequency response due to noise has zero mean and variance $\sigma_{n}^{2}/\sigma_{X}^{2}$.

The transfer function for the system, H(s), was estimated by taking the average of the difference FRFs across all pair-wise trial combinations (i, j),

$$H\left(s\right)≅FRF\left(s\right)=\frac{1}{M}\sum_{i=1}^{N}\sum_{\begin{array}{c} j=2\\ j>i \end{array}}^{N}\frac{\theta_{a_{i}}\left(s\right)-\theta_{a_{j}}\left(s\right)}{X_{i}\left(s\right)-X_{j}\left(s\right)}$$

where M is the number of pairwise trial combinations and the contribution of the (zero-mean) noise spectrum decreases as the inverse square root of M.

## Authors’ original submitted files for images

Below are the links to the authors’ original submitted files for images. Authors’ original file for figure 1 Authors’ original file for figure 2 Authors’ original file for figure 3 Authors’ original file for figure 4 Authors’ original file for figure 5 Authors’ original file for figure 6 Authors’ original file for figure 7 Authors’ original file for figure 8 Authors’ original file for figure 9 Authors’ original file for figure 10
