# Supplementary figures and images for: Intention tremor and deficits of sensory feedback control in multiple sclerosis: a pilot study
Source: J Neuroeng Rehabil. 2014 Dec 19;11:170. doi: 10.1186/1743-0003-11-170 (PMC4292988; doi:10.1186/1743-0003-11-170)

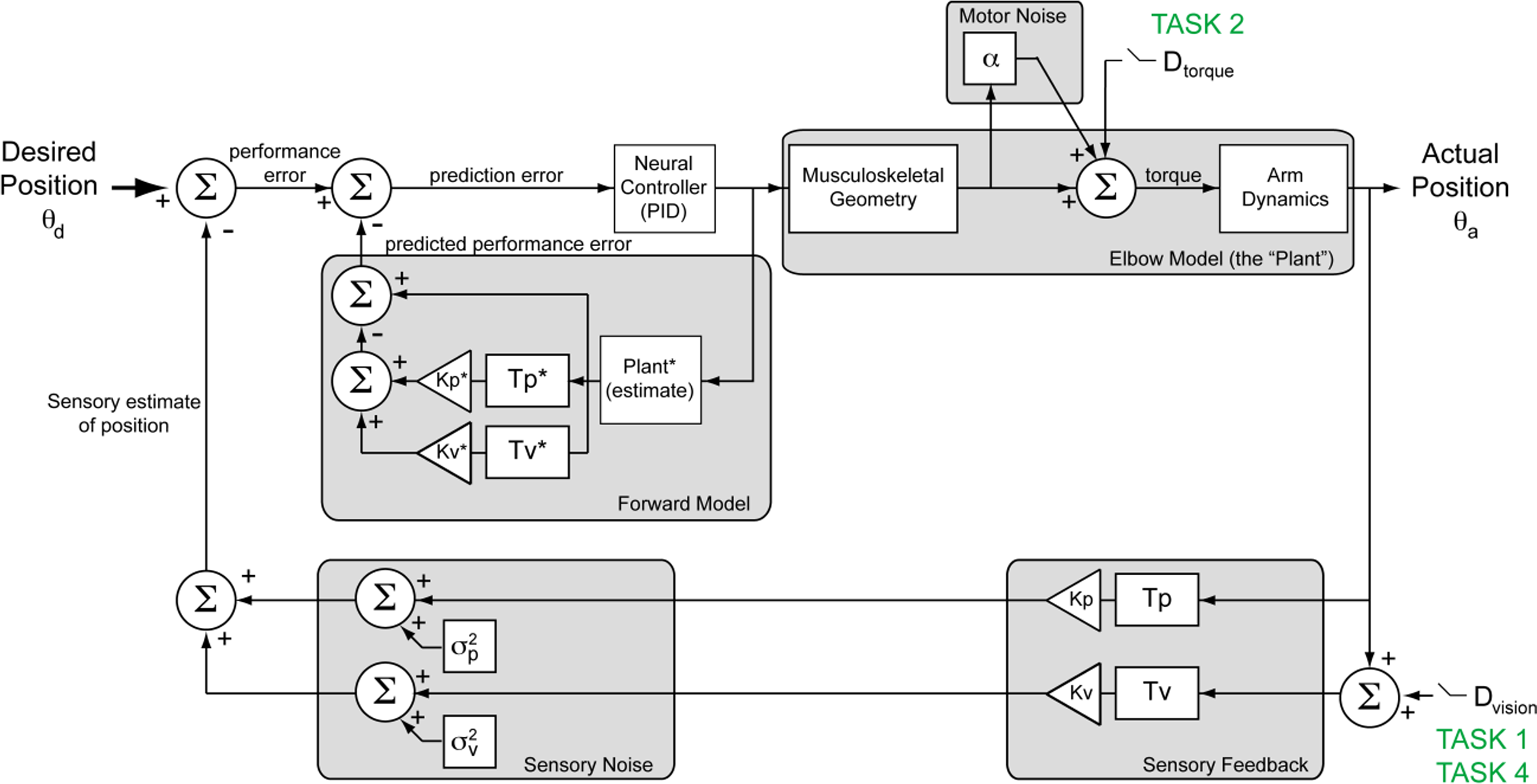

Supplement: Supplementary file 1 — Authors’ original file for figure 1 [file 12984_2014_689_MOESM1_ESM.tif]

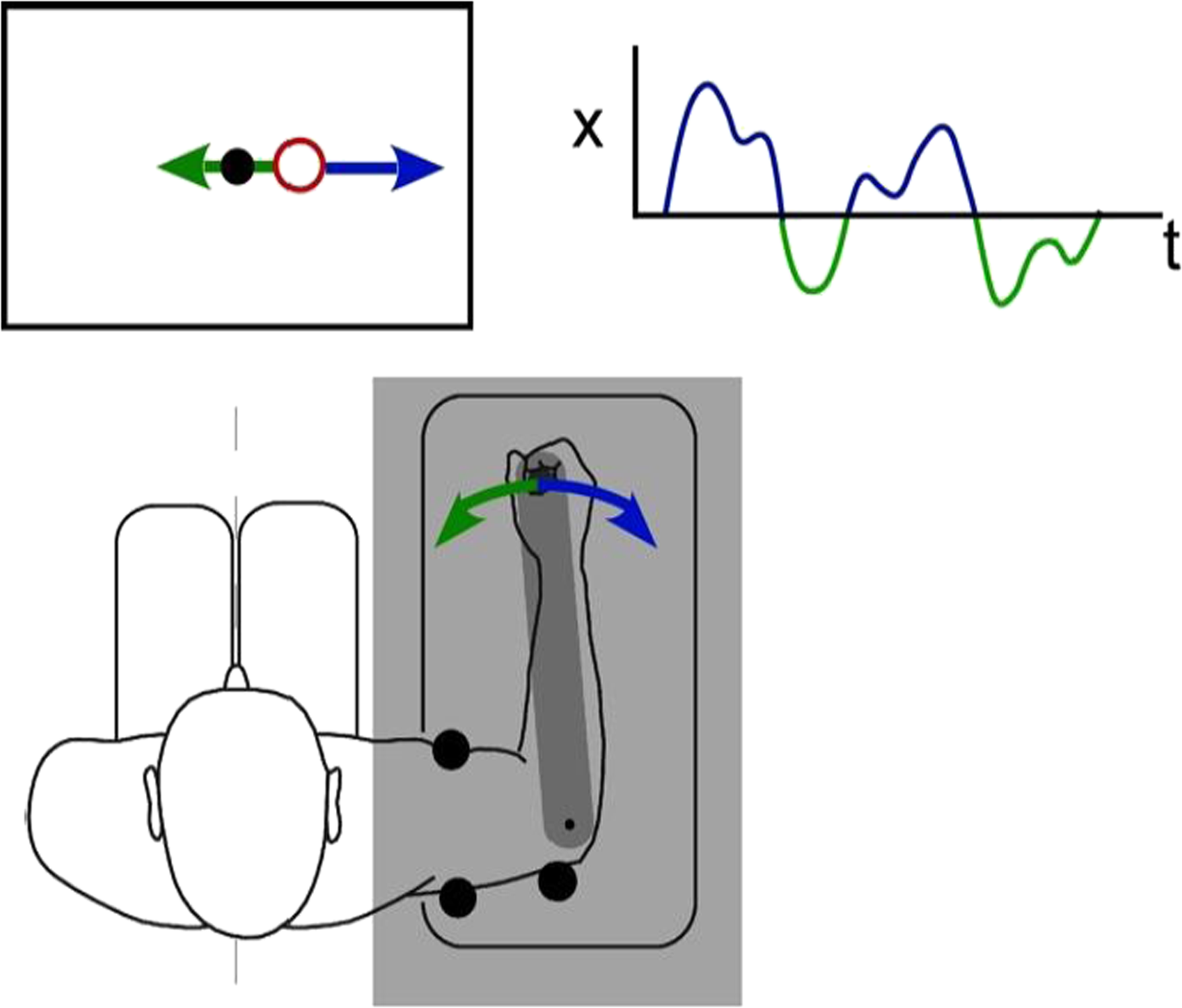

Supplement: Supplementary file 2 — Authors’ original file for figure 2 [file 12984_2014_689_MOESM2_ESM.tif]

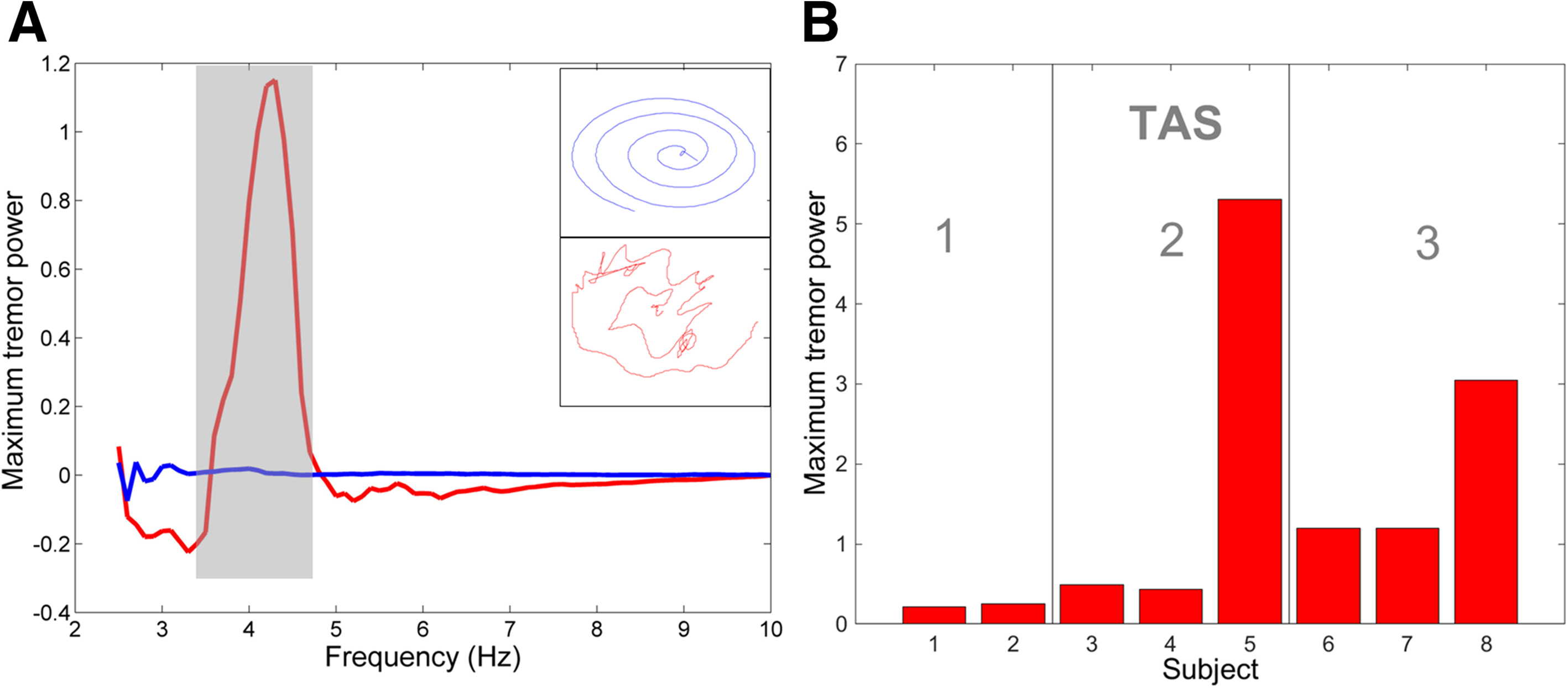

Supplement: Supplementary file 3 — Authors’ original file for figure 3 [file 12984_2014_689_MOESM3_ESM.tiff]

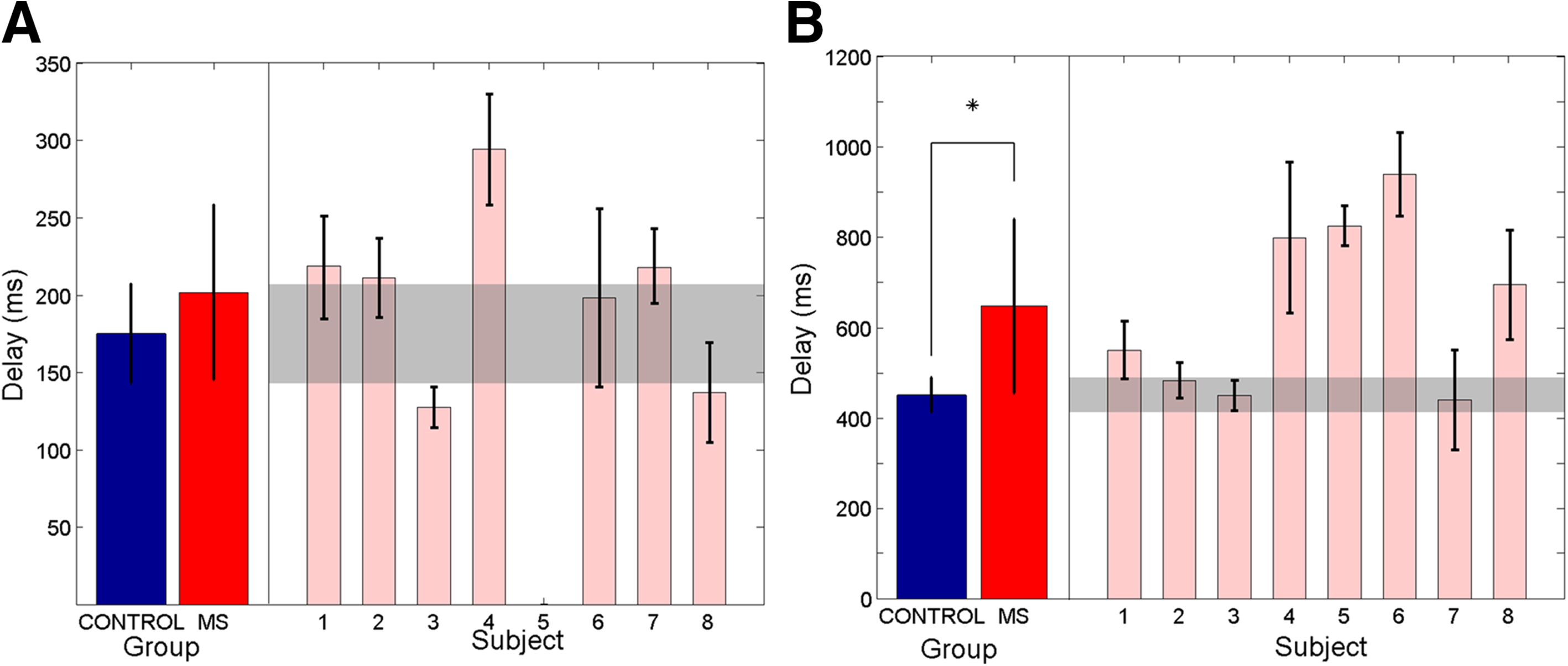

Supplement: Supplementary file 4 — Authors’ original file for figure 4 [file 12984_2014_689_MOESM4_ESM.tiff]

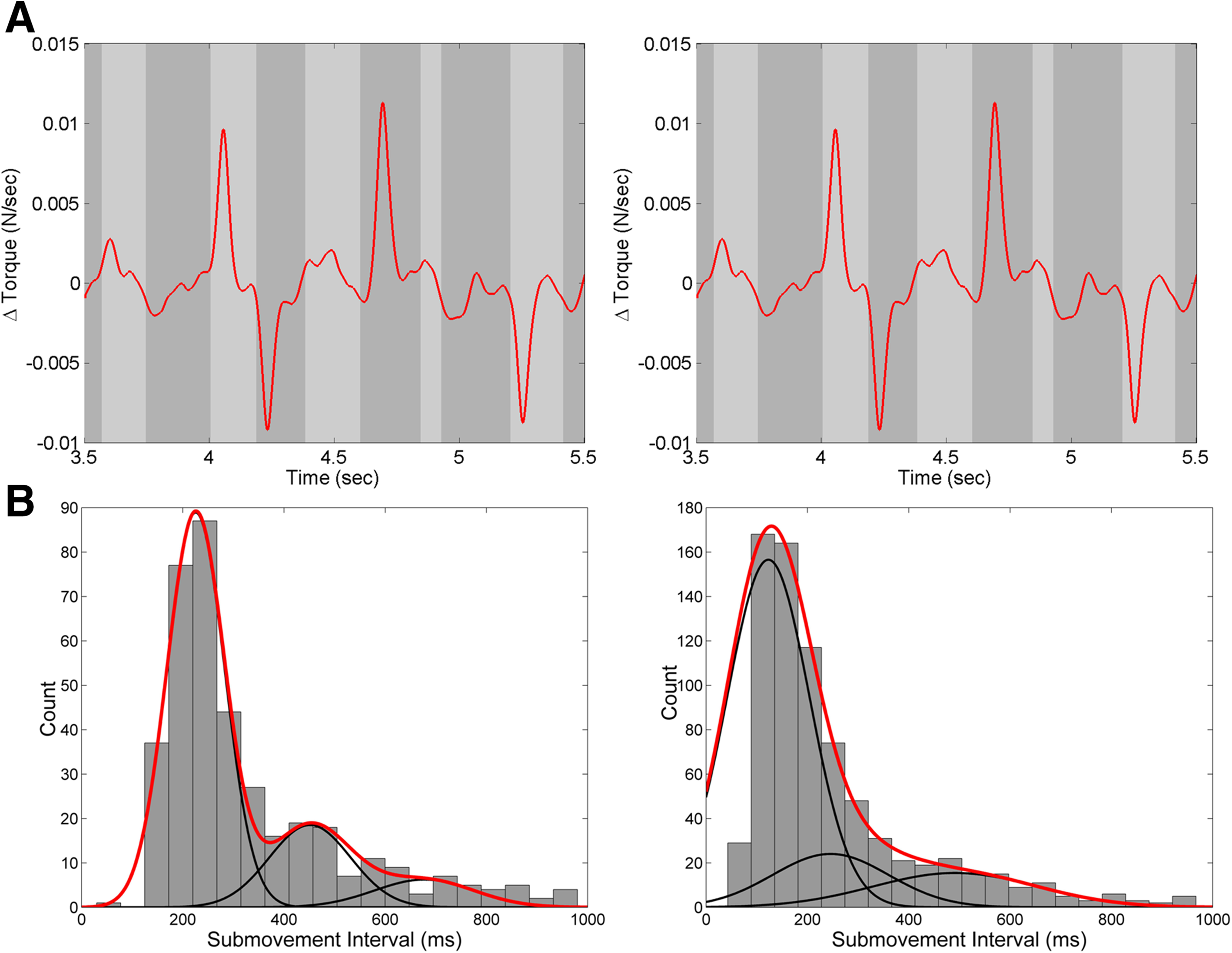

Supplement: Supplementary file 5 — Authors’ original file for figure 5 [file 12984_2014_689_MOESM5_ESM.tiff]

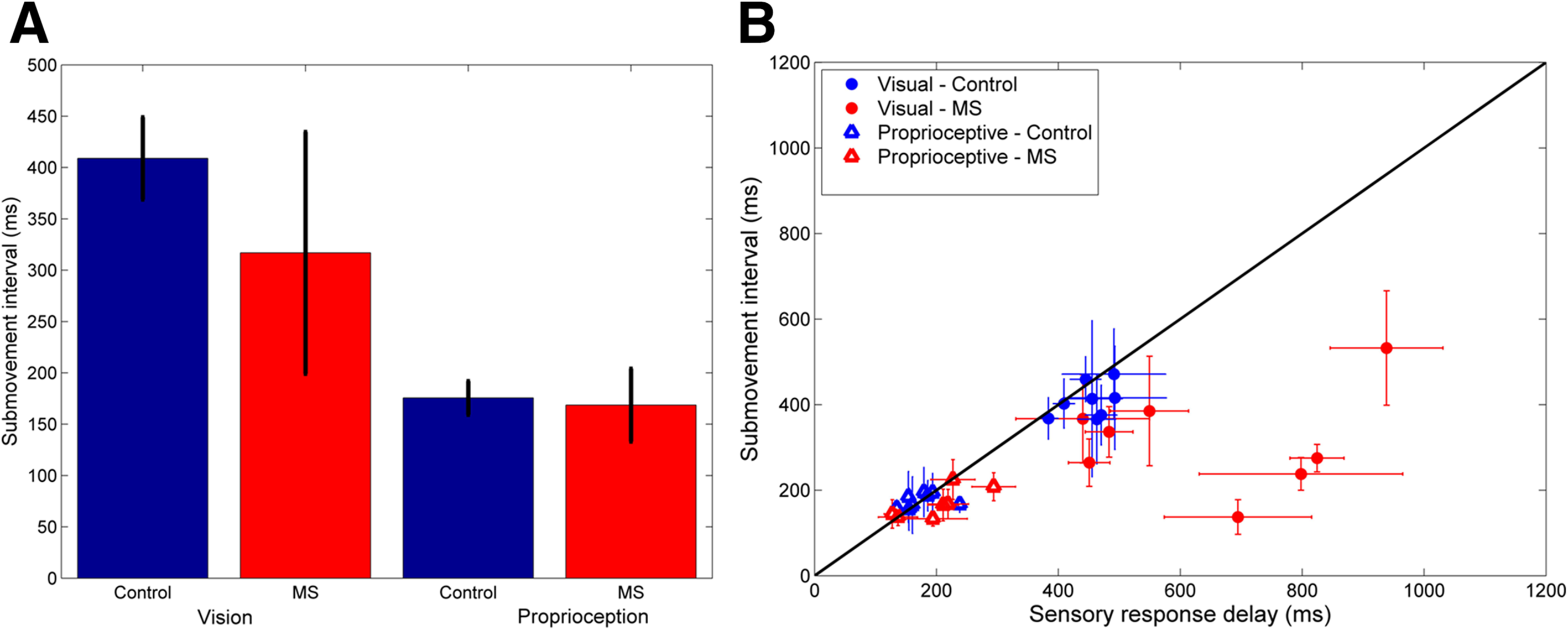

Supplement: Supplementary file 6 — Authors’ original file for figure 6 [file 12984_2014_689_MOESM6_ESM.tiff]

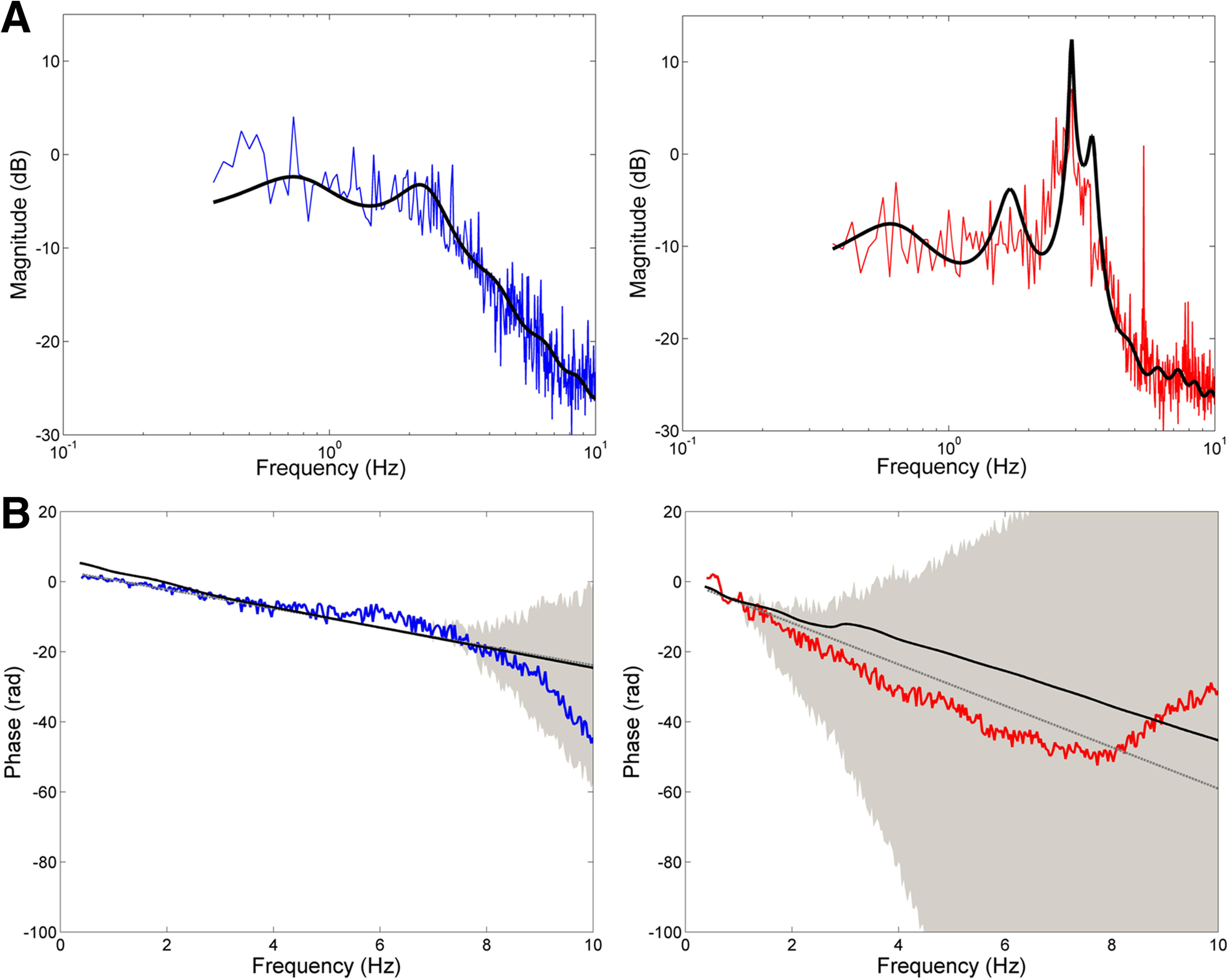

Supplement: Supplementary file 7 — Authors’ original file for figure 7 [file 12984_2014_689_MOESM7_ESM.tiff]

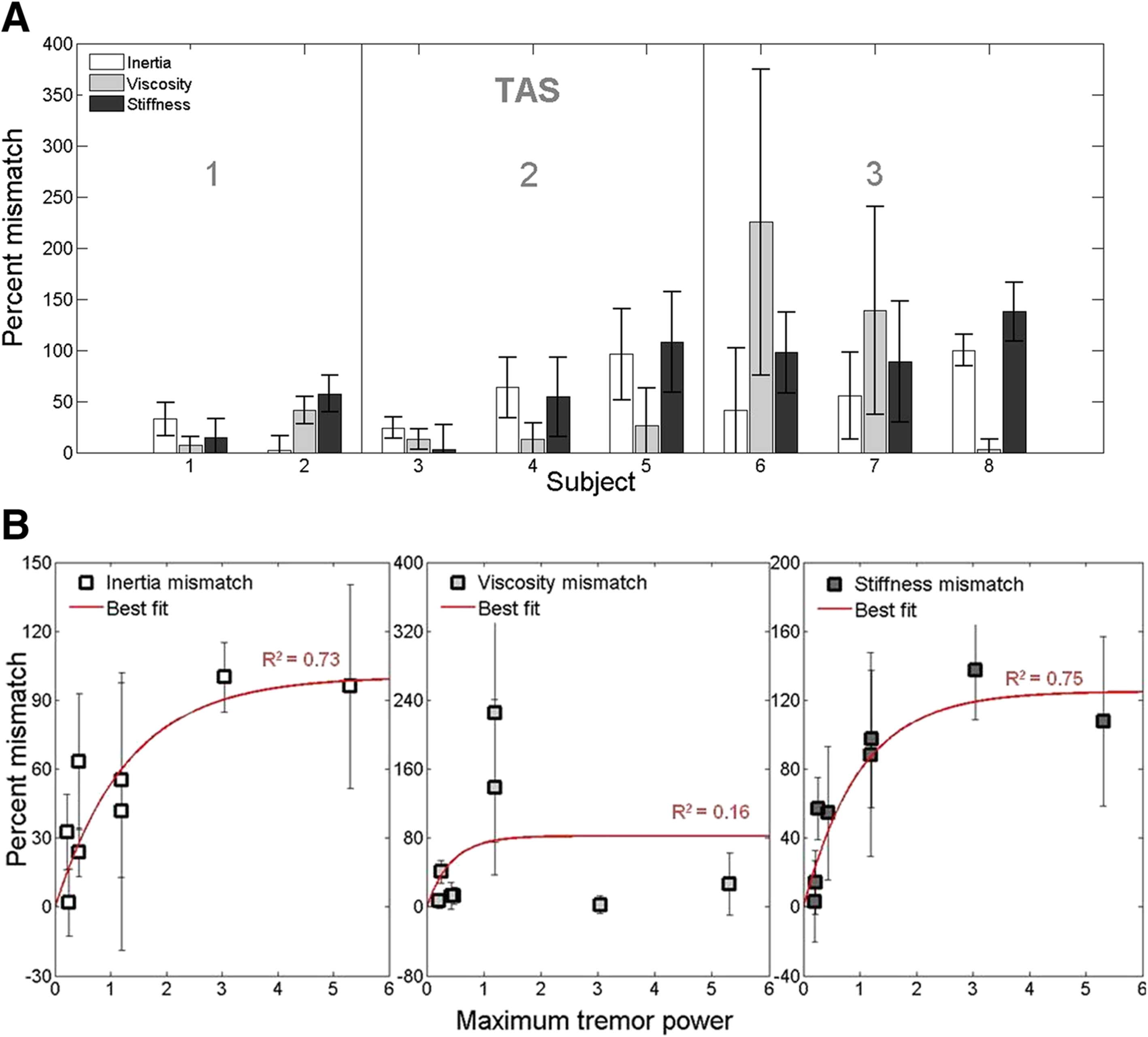

Supplement: Supplementary file 8 — Authors’ original file for figure 8 [file 12984_2014_689_MOESM8_ESM.tiff]

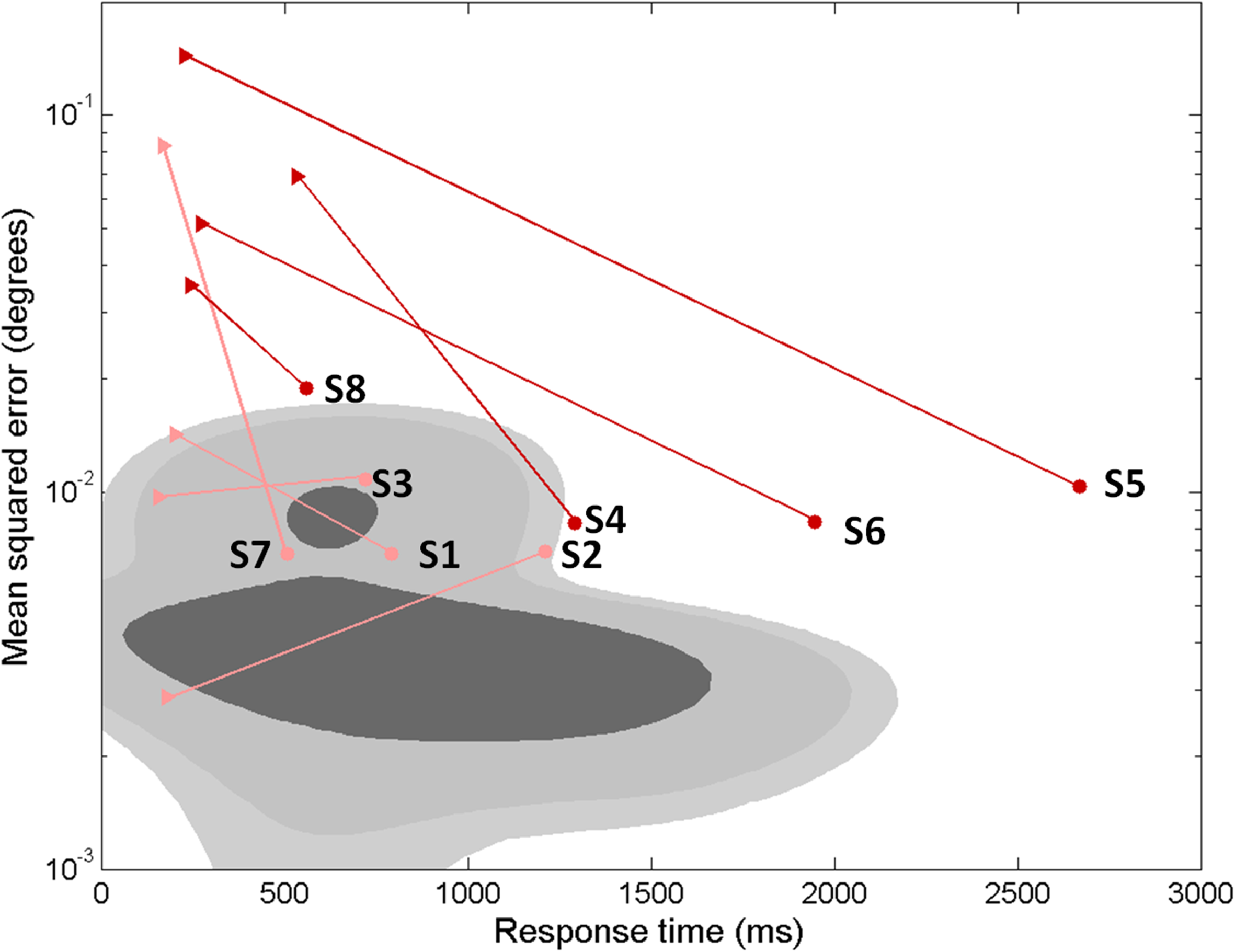

Supplement: Supplementary file 9 — Authors’ original file for figure 9 [file 12984_2014_689_MOESM9_ESM.tiff]

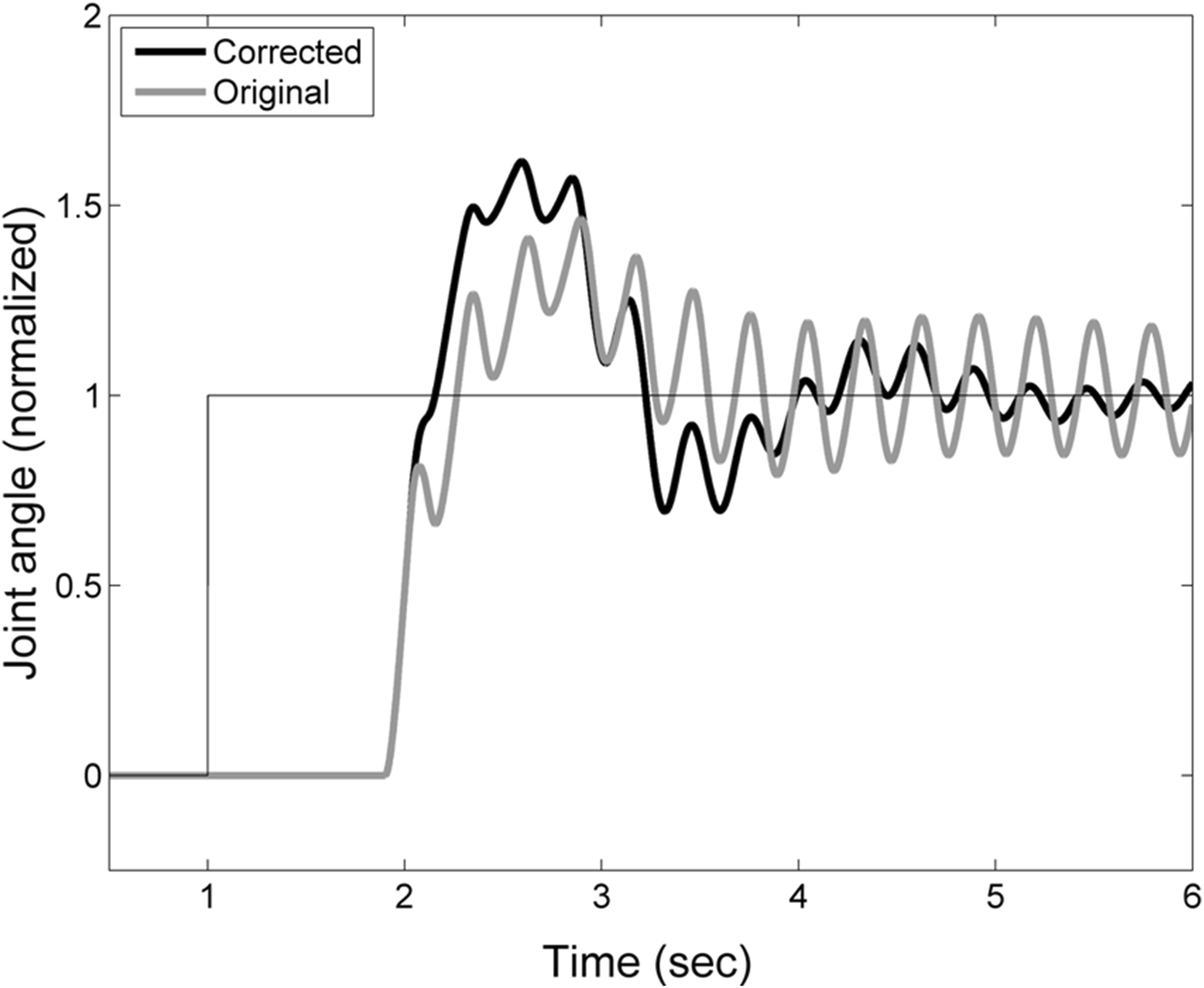

Supplement: Supplementary file 10 — Authors’ original file for figure 10 [file 12984_2014_689_MOESM10_ESM.tiff]
